# Chromium Hyper-Tolerant *Bacillus* sp. MH778713 Assists Phytoremediation of Heavy Metals by Mesquite Trees (*Prosopis laevigata*)

**DOI:** 10.3389/fmicb.2019.01833

**Published:** 2019-08-13

**Authors:** Verónica Ramírez, Antonino Baez, Primavera López, Rocío Bustillos, Miguel Ángel Villalobos, Ricardo Carreño, José Luis Contreras, Jesús Muñoz-Rojas, Luis Ernesto Fuentes, Javier Martínez, José Antonio Munive

**Affiliations:** ^1^Centro de Investigaciones en Ciencias Microbiológicas, Instituto de Ciencias, Benemérita Universidad Autónoma de Puebla, Puebla, Mexico; ^2^Centro de Investigaciones en Dispositivos Semiconductores, Instituto de Ciencias, Benemérita Universidad Autónoma de Puebla, Puebla, Mexico; ^3^Centro de Investigación en Biotecnología Aplicada, Instituto Politécnico Nacional, Tlaxcala, Mexico; ^4^Facultad de Arquitectura, Benemérita Universidad Autónoma de Puebla, Puebla, Mexico

**Keywords:** heavy metal, tolerance, phytoremediation, *Bacillus*, *Prosopis* (mesquite), chromium (VI)

## Abstract

Heavy metal accumulation in mesquite trees (*Prosopis laevigata*) growing in aluminum, titanium, chromium and zirconium-polluted soils of a semi-arid region in Mexico was investigated using wavelength dispersive X-ray fluorescence analysis. The results showed that *P. laevigata* trees can hyper accumulate up to 4100 mg/kg of Al, 14000 mg/kg of Fe, 1600 mg/kg of Ti, 2500 mg/kg of Zn, but not chromium, regarding high chromium concentrations found in soils (435 mg/kg). Since plant-associated microorganism can modulate phytoremediation efficiency, the biodiversity of *P. laevigata* associated bacteria was studied. Eighty-eight isolates from *P. laevigata* nodules were obtained; all isolates tolerated high concentrations of Al, Fe, Zn and Cr *in vitro*. The top-six chromium tolerant strains were identified by 16S rRNA sequence analysis as belonging to genus *Bacillus*. *Bacillus* sp. MH778713, close to *Bacillus cereus* group, showed to be the most resistant strain, tolerating up to 15000 mg/L Cr (VI) and 10000 mg/L of Al. Regarding the bioaccumulation traits, *Bacillus* sp. MH778713 accumulated up to 100 mg Cr(VI)/g of cells when it was exposed to 1474 mg/L of Cr VI. To assess *Bacillus* sp. MH778713 ability to assist *Prosopis laevigata* phytoremediation; twenty plants were inoculated or non-inoculated with *Bacillus* sp. MH778713 and grown in nitrogen-free Jensen’s medium added with 0, 10 and 25 mg/L of Cr(VI). Only plants inoculated with *Bacillus* sp. grew in the presence of chromium showing the ability of this strain to assist chromium phytoremediation. *P. laevigata* and *Bacillus* spp. may be considered as good candidates for soil restoration of arid and semiarid sites contaminated with heavy metals.

## Introduction

With the onset of fast-developing industries, farming, energy stations and the inappropriate waste disposal practices, soil and water have been deeply contaminated with organic compounds and heavy metals with permanent toxic effects on ecosystems and human health (Kalbitz and Wennrich, 1998; Claret et al., 2011; Mahar et al., 2016). In the arid and semiarid zones, the major sources of heavy metals as contaminants are the use of pesticides for plant protection or vector control, large-scale crop protection campaigns, mining and inadequate management of industrials wastes. The vulnerability of ecosystems to contaminants is closely related to water flow (Everts, 1997), so that the low rainfall, characteristic of arid and semiarid zones, make these ecosystems easily affected by the presence of toxic elements. Heavy metal is the generic name given to the group of elements with an atomic density greater than 6 g/cm^3^, ubiquitous in the Earth’s crust. These metals include lead (Pb), cadmium (Cd), nickel (Ni), cobalt (Co), iron (Fe), zinc (Zn), chromium (Cr), silver (Ag), titanium (Ti) and copper (Cu). Chromium is widely used in electroplating, leather tanning, textile dyeing, and metal processing industries. In metal cleaning, plating, and metal processing industries, chromium concentration of effluents can reach 20000–75000, 15000–52000 and 100000–270000 ng/mL (Gowd and Govil, 2018). The most stable and abundant forms of chromium in nature are chromium (III) and chromium (VI), the latter being the most toxic (Kotas and Stasicka, 2000).

Conventional soil remediation techniques like excavation, landfill, soil leaching/acid extraction and soil washing, are inadequate, costly and often involve the storage of contaminated effluents in designated areas, postponing rather than solving the problem (Ullah et al., 2015; Wang et al., 2017). The use of plants in metal extraction (phytoremediation) has appeared as a safe and cost-effective alternative in the removal of heavy metal excess from soil and water (Chaney et al., 1997; Glass, 1999; Mulligan et al., 2001). There are two types of plants suitable for phytoremediation, the biomass producers and the metal hyperaccumulators; both remove heavy metal from the soil through the roots with subsequent transport to aerial parts. Hyperaccumulator species can accumulate one or more inorganic toxic element up to 100-fold higher than other species growing under the same conditions (Cappa and Pilon-Smits, 2014). Because of their capacity to act as efficient interceptors and accumulators of chemicals, vegetation species are widely employed as passive monitors in contaminated areas by heavy metals (Álvarez et al., 2003; Marques et al., 2003).

*Prosopis* spp., commonly known as mesquite, can tolerate drought, alkaline pH, extreme temperatures and high concentrations of salt. Mesquite is a native flora in areas with water deficit where it is a valuable economic resource. These trees can produce a large amount of biomass until 14.5 T ha^–1^yr^–1^ in hot and arid conditions (Felker et al., 1983). Assays have demonstrated the ability of *Prosopis* to tolerate, translocate, and hyper accumulate heavy metals such as Pb (Jayaram and Prasad, 2009), As (Aldrich et al., 2003, 2007; Gardea-Torresdey et al., 2004; Mokgalaka et al., 2008), Cu (Nivethitha et al., 2002; Zappala et al., 2014), Zn (Nivethitha et al., 2002) and the capability to reduce Cr (VI) to Cr (III) (Aldrich et al., 2003). *Prosopis juliflora*, a multipurpose perennial tree native to South America (Sajjad et al., 2012), has been studied as a possible bioindicator of soil pollution (Senthilkumar et al., 2005). Therefore, selecting the appropriate plant species is fundamental for a successful phytoremediation process.

Several microorganisms have the exceptional ability to adapt to metal-polluted environments, where higher organisms are unable to occur. These microorganisms have developed the capabilities to protect themselves from heavy metal toxicity by various mechanisms such as adsorption, uptake, methylation, oxidation, and reduction. The reduction of the highly soluble and toxic chromium (VI) to the less soluble and less toxic chromium (III) is carried out by *Acinetobacter* sp. (Francisco et al., 2002), *Arthrobacter* sp. (Megharaj et al., 2003), *Pseudomonas* sp. (Rajkumar and Nagendran, 2005), *Serratia marcescens* (Campos et al., 2005), *Ochrobactrum* sp. (Thacker and Madamwar, 2005), *Desulfovibrio vulgaris* (Goulhen et al., 2006), *Cellulomonas* sp. (Viamajala et al., 2007), and *Bacillus* sp. (Elangovan et al., 2006; Rehman et al., 2008). Thus, microorganisms by themselves can be used to remediate metal-contaminated soils (Park et al., 2011). The synergism of plants and their rhizosphere-associated microorganisms can be used to enhance the removal of toxic contaminants from the environment, improving phytoremediation processes (Ashraf et al., 2017). Microorganisms appear to be beneficial in phytoremediation by improving metal solubility because of the production of organic acids, by modifying the soil structure because of the secretion of polysaccharides, or by producing plant growth-promoting substances (Wang et al., 2017). Phytoremediation could be significantly boosted by the inoculation of metal-resistant beneficial microorganism due to the potential of microbes to bio-accumulate metals from contaminated soils enhancing the metal mobilization by plants (Mench and Martin, 1991; Ullah et al., 2015). In fact, inoculation of plants with mycorrhizal or plant growth-promoting bacteria have shown augmented positive effects on phytoremediation experiments (Dimkpa et al., 2009; Hou et al., 2015; Xun et al., 2015).

The interactions of metal tolerant microorganisms and hyper-accumulating plants working together in natural wildlife habitat are scarcely studied. This work aimed to describe the levels of heavy metals prevalent in rhizospheric and non-rhizospheric soils of a semiarid site, and the levels of accumulation in different plant tissues of mesquite trees (*Prosopis laevigata*) grown in an area contaminated with heavy metals of Nexapa river in Chietla, Puebla, Mexico. This river is contaminated with high concentrations of metals such as Fe, Al, Pb, Cd, Zn and Cr (Pérez et al., 2018). Beside to describe the phytoremediation ability of mesquite trees grown in their natural wildlife habitat, the microbial diversity inside the roots nodule of mesquites trees is described. 88 endophytic bacteria isolated from the root nodules of *Prosopis laevigata* are characterized and their chromium biosorption properties and phytoremediation assistance are described.

## Materials and Methods

### Sampling Location

The site of this field study was in the region of Nexapa River Chietla, Puebla, Mexico, 18°31′27. 5^″^N 98°35′02. 0^″^W.1000 MASL where soils and *Prosopis laevigata* trees were sampled. The average annual precipitation in this area is 806.7 mm (Martínez-Pérez et al., 2012) corresponding to the superior threshold for semi-arid lands (Fao Conservation Guide 20, 1989). All young *P. laevigata* trees were nodulated, while adult trees were not, or nodules were not detectable by our observation method. The sheaths and leaves samples were taken at the main branches at 2 meters, above the floor; the stems samples were taken at 1.2 meters above of the ground; the main tree roots were sampled at 50 cm depth for nodules collection; rhizospheric soils were taken at 20 cm depth (Ernst, 1995).

### Strains and Culture Conditions

Eighty-eight bacterial strains were isolated from *P. laevigata* nodules on Yeast Mannitol Agar (YMA) (Vincent, 1970). Strains were grown on YMA at 28°C. They were checked for purity by repeated streaking and by microscopic examination. The strains were kept in 20% (v/v) glycerol at −80°C.

### Nodulation Test

All isolated strains were evaluated for their ability to induce nodules on *Macroptilium atropurpureum* plants, previously reported to be one of the most promiscuous legume hosts in the group of rhizobia (Somasegaran and Hoben, 1994), and only MH778712 and MH778713 isolates were further assayed for nodulation capability in their natural host (*P. laevigata*). *Prosopis* or *Macroptilium* seeds were rinsed in water and sterilized by immersion in concentrated sulfuric acid (98%) for 20 min, rinsed with sterile water, and then left in water overnight. Seeds were placed onto water agar (0.75% w/v) in Petri dishes until germination before transferring them to agar slants (20 ml of agar in 50 ml tube) with Jensen’s N-free medium (Somasegaran and Hoben, 1994). A single colony of each isolate was picked from YMA plates and grown aerobically in yeast extract-mannitol broth (YM) (at 130 rpm) and 28°C. Cells were harvested by centrifugation at 5000 rpm for 10 min at 4°C. Each seedling was inoculated with 1 × 10^6^ UFC suspended in 1 ml of sterile distilled water. For each plant species tested, controls consisted of non-inoculated seedlings, either supplied with mineral nitrogen (as 0.1% KNO_3_ in nutrient solution) or grown without nitrogen (Somasegaran and Hoben, 1994). Plants were incubated at 20°C with a 16-h light, 8-h dark cycle. Tubes were observed for root nodule formation 3 to 4 weeks after inoculation, the nodulation test was done in triplicate for each isolate.

### Bacterial Endospore Stain on Schaeffer-Fulton

Bacterial strains isolated from *P. laevigata* nodules were stained with malaquite green according to the method of Schaeffer and Fulton for endospore observation (Schaeffer and Fulton, 1933).

### Amplification of 16S rRNA Gene

Total DNA was extracted from overnight cultures at 28°C in 15 ml of YM broth using the *Wizard Genomic DNA Purification Kit* (Promega Corporation, United States), according to the manufacturer’s protocol, and stored at 4°C. Amplification of 16S rRNA gene was carried out in a 2400 GeneAmp PCR Systems^®^ Perkin Elmer thermocycler. A PCR amplification product of 16S rRNA gene was obtained for each strain using universal primers UN27F (5′-TAGAGTTTGATCCTGGCTCAG-3′) and UN1392R (5′-CAGGGGCGGTGTGTACA-3′) (Morales et al., 2011) in a reaction volume of 25 μl using the Phusion^®^ High-Fidelity DNA Polymerase following the manufacturer’s instructions. The conditions were as follows: an initial activation step at 95°C for 3 min, followed by 26 cycles comprising denaturation at 94°C for 30 s, primer annealing at 57°C for 1 min, primer extension at 72°C for 70 s and finally extension at 72°C for 10 min. PCR products were purified with QIAquick PCR purification kit (QIAGEN GmbH, Hilden, Germany), and visualized under ultraviolet light after staining with ethidium bromide. In all cases, the amplification product size of 1500 bp was determined using the GeneRuler 1kb DNA Ladder (Thermo Fisher Scientific Inc., NYSE:TMO). Sequencing was performed using the sequencing unit service of the Biotechnology Institute (UNAM) (Applied Biosystems 3730XL DNA sequencer). Consensus sequences were obtained using the AutoAssembler software (Applied Biosystems).

### Sequence Alignment and Phylogenetic Analyses

The obtained 16S rRNA sequences were compared with those of strains belonging to the genus *Bacillus* retrieved from the Genbank/EMBL/DDBJ databases. Nucleotide sequence alignments were made using CLUSTAL_X (Thompson, 1997) and corrected manually using GeneDoc (Nicholas and Nicholas, 1997). The evolutionary history was inferred by using the Maximum Likelihood analyses performed with MEGA version 7 (Tamura et al., 2011; Kumar et al., 2016). ML analyses were performed using the Kimura 2-parameter model and were performed with 2000 bootstrap replications. Trees were visualized using MEGA version 7 (Tamura et al., 2011; Kumar et al., 2016).

### WDXRF (Wavelength Dispersive X-Ray Fluorescence) Assay

Samples of sheath, leaf, stem and root from 20 different mesquite trees were collected. These samples were dried and cut into 1 cm fragments, crushed and calcined at 500°C for 44 h, the ashes were sieved, and wax was added as a binder to prepare the composed samples. Twenty samples of rhizospheric soil, non-*rhizospheric* soil and sediment were also collected. Samples from each soil type were mixed, sieved, and 10 g of each soil sample were placed at 500°C for 24 h. Ashes were also sieved, and wax added as a binder to prepare the samples. All the samples were processed in a manual press and placed in an aluminum matrix. The quantitative analysis of the multi-elements was performed according to ISO9516-1: 2003 using the QUANT-EXPRESS method (Fundamental parameters) in the range of sodium (Na) to Uranium (U) in the wavelength dispersive x-ray fluorescence spectrometer of 1 kw Bruker model S8 TIGER, which has a detector of scintillation (Heavy elements) and flow (light elements), Rhodium (Rh) tube as an X-ray source and high-pressure goniometer for theta and 2 theta angles, with a sensitivity of up to 20 mg/kg. The total analysis time for each sample was 20 min. Which comprises a unique multipurpose prepared calibration using certified standards (STG2).

### Evaluation of Chromium Tolerance

One percent (v/v) of the log-phase culture was inoculated in YMA plates added with different concentrations of Cr (VI) (K_2_CrO_4_) (10000-15000 mg/L) and incubated at 28°C for 24 h (Corral et al., 2012). The minimal inhibitory concentration (MIC) of Cr (VI) at which no colony growth was observed, was determined using the Massive Stamping Drop Plate method, based on serial dilutions of the liquid samples (McLean et al., 2000). The chrome tolerance evaluation was done in triplicate for each isolate and concentration tested.

### Biosorption Assays

A standard curve for the reaction between chromium and its binding dye, 1,5 diphenyl carbazide was constructed to determine the chromium concentration in each sample. The diphenyl carbazide forms a purple complex with hexavalent chromium but not with trivalent chromium (Young et al., 2003). Biosorption of chromium assay was carried out in 500 ml Erlenmeyer flask containing 100 ml of Cr (VI) (K_2_CrO_4_, Sigma-Aldrich, 7789-00-6) at 55, 218, 664, 905, 968, 1094 and 1474 mg/L and 1 g of *Bacillus* sp. MH778713 cells (3 × 10^13^ CFU). To obtain 1 g of cells, overnight cultures of *Bacillus* sp. MH778713 grown in YM medium were centrifuged, supernatants were discharged, and pellets were used to prepare 1g of biomass for each assay. The Erlenmeyer flasks containing chromium and 1g of cells were incubated at 37°C and 130 rpm in an orbital shaker for 7 h. Every hour, 10 ml samples were removed from Erlenmeyer flasks, cells were separated by centrifugation at 5000 rpm for 10 min at 4°C and residual Cr (VI) concentration in the supernatant was determined using diphenyl carbazide method (Bankar et al., 2009) at 540 nm using a UV-VIS JENWAY 6305 spectrophotometer. After each measurement, cells were returned to the Erlenmeyer flasks keeping sterile conditions. At the beginning and end of the experiment, serial dilutions were made and 10 μL of cell suspension was plated in YMA to determine the CFU/g and cell survival as described by Schell and Beermann (2014). The biosorption assay was done in triplicate for each concentration evaluated (Schell and Beermann, 2014).

### Adsorption Isotherm

The biosorption capacities (q_eq_) at equilibrium were calculated as follows:

q=eq(C-0C)eqV/X

where C_0_ was the initial Cr (VI) concentration (mg^*^L^–1^); C_eq_ the Cr (VI) concentration at equilibrium (mg^*^L^–1^); V the volume of solution used (L); X was biosorbent mass (g^*^L^–1^).

The removal efficiency (%) was defined as the ratio of metal ion concentration at equilibrium to the initial metal ion concentration and was calculated as follows:

removal efficiency %=(C-0C)eq/C×0100

### Plant Chromium-Tolerance Assays

Seeds of *Prosopis laevigata* were scarified with concentrated sulfuric acid (98%) for 20 min in constant agitation. Scarified seeds were placed onto water agar (1%) for 3 days in the dark for germination and then transferred to bottles with nitrogen-free Jensen’s medium added with 0, 10 and 25 mg/L of Cr(VI) (Davies et al., 2002; Shanker et al., 2005). Twenty plants for each treatment were incubated at 20°C with 16-h light, 8-h dark cycle. Three days later, seedlings were inoculated with 1 × 10^6^ UFC/ml of *Bacillus* sp. MH778713 whose cell were previously washed. Two months later, after true leaves appear, the plant-growth promotion was evaluated in the presence and absence of Cr(VI). Controls consisted of seedlings, either inoculated or non-inoculated, in nitrogen-free Jensen’s medium.

## Results

The Wavelength Dispersive X-Ray Fluorescence (WDXRF) is a powerful tool for qualitative and quantitative elemental analysis. *Prosopis laevigata* wildlife trees grown in a heavy metal-polluted landform were analyzed for their heavy-metal bioaccumulation. Table 1 shows the heavy metals concentrations found in the soil next to the edge of Nexapa river (the main source of heavy-metal contamination of the explored site), rhizospheric and non-rhizospheric soils, plant tissues (root, stem, leaf, sheath), and reference values (as reported in the literature) (Marguí et al., 2007). Fe, Zn, Al, Cr, and Ti elements were found in high levels in rhizospheric and non-rhizospheric soils; Al, Cr, and Ti high-levels confirm the heavy-metal contamination of the terrain (Table 1). Other elements like Si, Ca, S, P, K, and Na, were detected but their concentrations fell inside the normal range. Plant tissue analysis of adult trees showed that *Prosopis laevigata* grown in wildlife habitat can hyperaccumulate up to 4.1 × 10^3^ mg/kg of Al, 1.4 × 10^4^ mg/kg of Fe, 1.6 × 10^3^ mg/kg of Ti and 2.5 × 10^3^ mg/kg of Zn; being sheaths the most hyperaccumulator tissue (Table 1). Remarkably, Cr was not detected in any tissue of the trees despite the high concentration of Cr found in rhizospheric (208 mg/kg) and non-rhizospheric (435 mg/kg) soils. Similar results of heavy-metal bioaccumulation were obtained from young *Prosopis* trees (Supplementary Table S1). Wavelength Dispersive X-Ray Fluorescence spectrums are shown in Supplementary Figures S1–S7).

**TABLE 1:** Heavy Metal content of sediment, soil and plant tissues from the Nexapa river region.

**Element**	**Sediment (mg/kg)**	**Non-rhizospheric soil (mg/kg)**	**Rhizospheric soil (mg/kg)**	**Root (mg/kg)**	**Stem (mg/kg)**	**Leaf (mg/kg)**	**Sheath (mg/kg)**	**R Plant (mg/kg)**	**R Soil (mg/kg)**
Al	5.29 × 10^4^	4.43 × 10^4^	6.18 × 10^4^	4.1 × 10^3^	–	3.4 × 10^3^	–	1 × 10^3^	1 × 10^5^
Fe	4.55 × 10^4^	4.36 × 10^4^	5.13 × 10^4^	9.6 × 10^3^	3.2 × 10^3^	4.3 × 10^3^	1.4 × 10^4^	1200	500
Ti	5 × 10^3^	4.9 × 10^3^	5.3 × 10^3^	<20	–	<20	1.6 × 10^3^	80	9 × 10^3^
Cr	862	435	208	<20	–	–	–	0.2	50
Zn	154	108	174	638	<20	928	2.5 × 10^3^	20	120
Cu	135	<20	66.9	729	–	–	765	1500	100
Ni	218	<20	–	<20	–	–	–	100	90
Zr	–	<20	–	–	–	–	47.5	12	500

*The concentration of metal is given in mg/kg of dry weight. Sediment mean samples of Nexapa river sediment, Non-rhizospheric soil mean soil samples taken 300 m away from Nexapa river, Rhizospheric soil mean samples of *Prosopis laevigata* rhizospheric soil of trees located 200 m away from Nexapa river. The WDXRF specters are shown in Supplementary Figures S1–S7. Reference values for heavy metals found in plants and soils are shown in columns “R Plant” and “R soil” in this table (Kabata-Pendias, 2010).*

*Prosopis laevigata* trees were the primary plant present in the heavy-metal contaminated site of this study, suggesting that *Prosopis* plant has special traits that allow it to grow in hostile soils. The plant-associated microorganisms could provide such special traits. To determine the diversity of root-nodule bacteria associated with *Prosopis laevigata*, nodules of young trees growing in nature were analyzed. *Prosopis* trees were uprooted and nodule inhabiting bacteria were isolated using standard procedures (Vincent, 1970). Eighty-eight bacterial strains were isolated from nodules and tested for nodulation capacity in the promiscuous legume *Macroptilium atropurpureum*. None of 88 bacterial strains was able to induce nodule formation.

**FIGURE 1:**
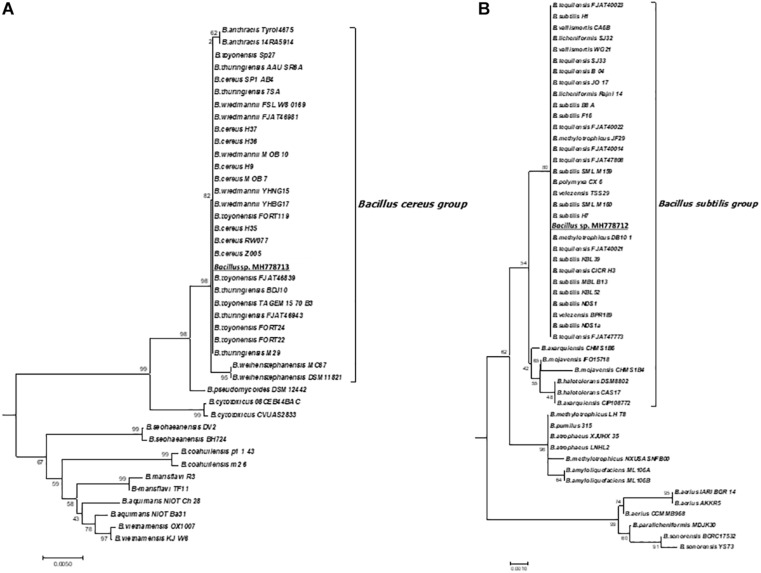
Maximum likelihood tree based on 16S rRNA gene sequences showing the relationships of *Bacillus* strains MH778713, MH778712 and other *Bacillus* species in the *Bacillus cereus* group **(A)** and *Bacillus subtilis* group **(B)**. The evolutionary history was inferred using the Neighbor-Joining method. The optimal tree with the sum of branch length = 1.82220521 is shown. The percentage of replicate trees in which the associated taxa clustered together in the bootstrap test (2000 replicates) are shown next to the branches. The tree is drawn to scale, with branch lengths in the same units as those of the evolutionary distances used to infer the phylogenetic tree. The evolutionary distances were computed using the Maximum Composite Likelihood method and are expressed as the units of the number of base substitutions per site. The analysis involved 378 nucleotide sequences. All positions containing gaps and missing data were eliminated. There were a total of 1114 positions in the final dataset. Evolutionary analyses were conducted in MEGA7.

Since the interaction of metal-tolerant bacteria with hyper-accumulating plants can improve phytoremediation, we looked for Fe-, Zn-, Cr-tolerant strains by growing each of the 88 nodule-isolated bacteria with increasing concentrations of each metal in YMA plates. All isolates grew with 500 mg/L of Fe, 19 isolates tolerated up to 1000 mg/L Fe but none of them stood at 1100 mg/L. Zn tolerance was also assayed; the 88 isolated strains grew with 300 mg/L, fifteen of them tolerated up to 2000 mg/L and no one endured 2100 mg/L of Zn. Regarding Cr (VI) tolerance, all isolates grew in the presence of 1500 mg/L Cr (VI) and six of them tolerated up to 10000 mg/L (Table 2). The MH778712 and MH778713 isolates grew at 10500 mg/L, being the MH778713 strain the only one able to tolerate up to 15000 mg/L Cr (Table 2). Considering the high aluminum concentrations in the soils, the hyper Cr-tolerant strain (*Bacillus* sp. MH778713) was also challenged with aluminum, being able to tolerate up to 10000 mg/L (Supplementary Figure S8). The 16S rRNA sequence analysis of the top-six Cr (VI) tolerant strains isolated from *Prosopis laevigata* nodules indicated that all-six strains belong to the genus *Bacillus.* Furthermore, the *Bacillus* genus was corroborated by endospore observation through Schaeffer-Fulton staining. 16S rRNA-based phylogenies indicated that MH778713 and MH778712 strains are close to *Bacillus cereus* and *Bacillus subtilis* groups (Figure 1). Finally, the ability of isolates MH778713 and MH778712 to nodulate *Prosopis laevigata* was tested. Both strains failed to induce nodule in their native host *Prosopis laevigata*.

**TABLE 2:** Tolerance of *Bacillus* strains to Cr(VI) by serial dilution using the Massive Stamping Drop Plate method.

***Bacillus* strains**	**UFC/ml at 10 000 mg/L**	**UFC/ml at 10 500 mg/L**	**UFC/ml at 11 000 mg/L**	**UFC/ml at 11 500 mg/L**	**UFC/ml at 12 000 mg/L**	**UFC/ml at 15 000 mg/L**
LEM1004	2 × 10^5^	1 × 10^4^	NG	NG	NG	NG
LEM1054	2 × 10^5^	1 × 10^5^	NG	NG	NG	NG
LEM1080	6 × 10^4^	4 × 10^3^	NG	NG	NG	NG
MH778712	1 × 10^6^	2 × 10^5^	4 × 10^3^	2 × 10^3^	NG	NG
LEM1085	2 × 10^5^	NG	NG	NG	NG	NG
MH778713	2 × 10^9^	3 × 10^9^	1 × 10^9^	1.8 × 10^9^	1.4 × 10^9^	1.5 × 10^9^

*Bacteria were grown in YMA medium with different chromium concentrations and incubated at 30°C for 24 h. The growth of the top six tolerant strains are shown in this table. NG (No growth).*

**FIGURE 2:**
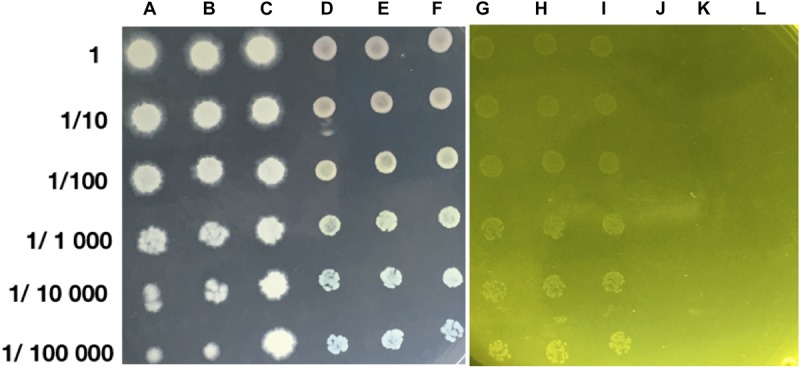
*Bacillus* sp. MH778713 **(A–C)** and *Pseudomonas putida* KT2440 **(D–F)** in YMA medium without Cr (VI). Cr (VI) tolerance of *Bacillus* sp. MH778713 plating in YMA with 15000 mg/L of Cr (VI) using the Massive Stamping Drop Plate method **(G–I)** comparing with Pseudomonas putida KT2440 **(J–L)**.

**FIGURE 3:**
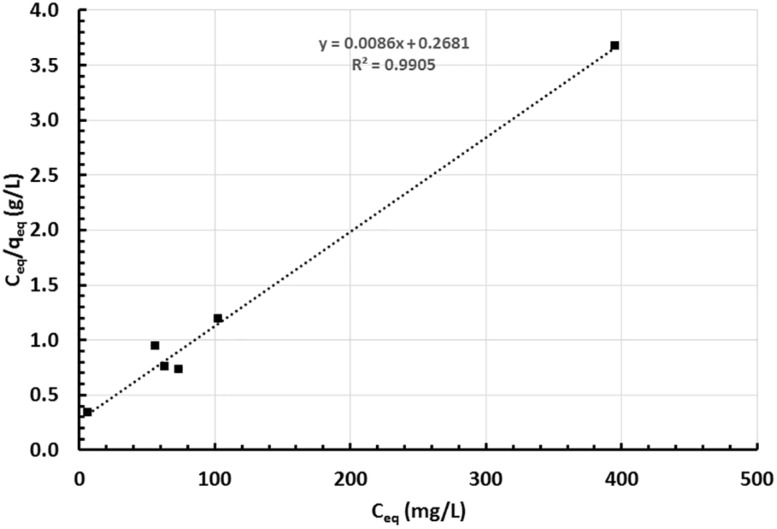
Langmuir adsorption isotherm of chromium (VI) by *Bacillus* sp. MH778713 using 1,5-Diphenyl carbazide method at 37°C, 130 rpm, and *t* = 0–420 min. Data represent the average of triplicate experiments.

**FIGURE 4:**
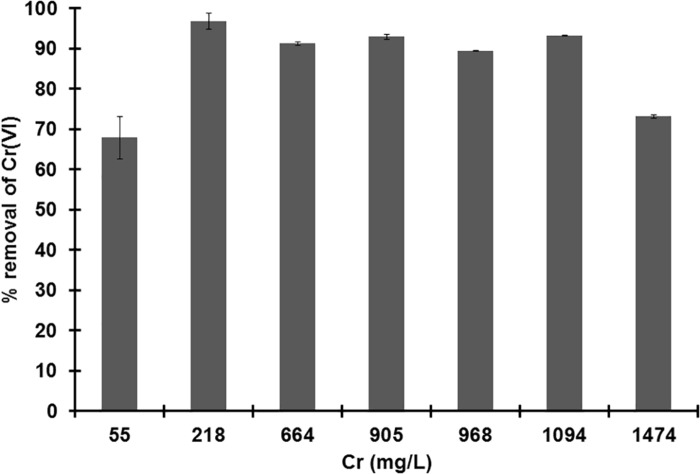
Percentage of chromium (VI) removal by *Bacillus* sp. MH778713 from supernatant after 7 h of exposure at different Cr (VI) concentrations. *Bacillus* sp. MH778713 is capable to remove more than 95% of environmental chromium (VI) at 218 mg/L. Data represent the average of triplicate experiments.

The growth of strain MH778713 was compared with *Pseudomonas putida* KT2440 in YMA plates without and with 15000 mg/L Cr (VI) (Figure 2). Both strains grew in YMA plates without chromium but only our isolate MH778713 grew with 15000 mg/L of Cr and reached 1.5 × 10^9^ CFU/mL (Figure 2 lines G–I). Even *P. putida* KT2440 is known by its metal-tolerance qualities, it did not grow in the presence of 15000 mg/L Cr (Figure 2 lines J–L). To determine chromium-biosorption capability of *Bacillus* sp.MH778713 a colorimetric assay was performed using 1,5 diphenyl carbazide (Supplementary Figure S9). Biosorption equilibrium data were analyzed by fitting experimental data into the Langmuir isotherm model (Figure 3) (Bankar et al., 2009). Thus, *Bacillus* sp. MH778713 showed a sorbent capacity (A_s_) of 116 mg of Cr (VI)/g of cell, which was higher than it reported for *Yarrowia lipolytica* (Bankar et al., 2009). The survival of *Bacillus* cells after 7 h exposure to this chromium concentration was 85% (3 × 10^11^ CFU/g) (Supplementary Figure S10). In all cases, *Bacillus* strain MH778713 was capable to absorb more than 65% of environmental chromium (VI), with the maximum percentage of biosorption (95%) at 218 mg/L (Figure 4 and Supplementary Figure S9). A linear relationship between initial chromium concentration and the maximum metal-uptake value was obtained (Supplementary Figure S9). This biosorption capability of *Bacillus* sp. MH778713 might contribute to the elimination of chromium from *Prosopis* tissues (Table 1).

**FIGURE 5:**
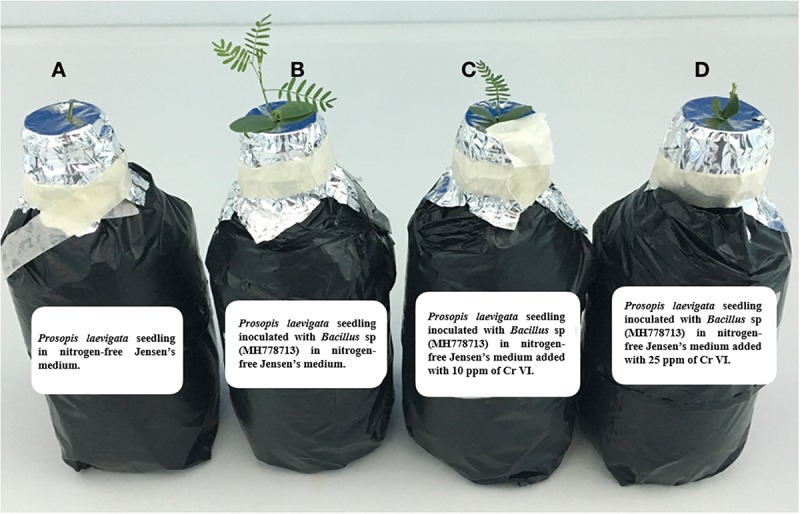
Chromium (VI) tolerance assay of *Prosopis laevigata* seedlings inoculated with *Bacillus* sp. MH778713 grown in a hydroponic system. **(A)** Uninoculated *P. laevigata* seedlings in nitrogen-free Jensen’s medium as negative controls. **(B)** *P. laevigata* seedlings inoculated with *Bacillus* sp. MH778713 in nitrogen-free Jensen’s medium as a control. **(C)** *P. laevigata* seedlings inoculated with *Bacillus* sp. MH778713 in nitrogen-free Jensen’s medium added with 10 mg/L of Cr(VI). **(D)** *P. laevigata* seedlings inoculated with *Bacillus* sp. MH778713 in nitrogen-free Jensen’s medium added with 25 mg/L of Cr(VI). This single-plant picture is a representative image of the response of 20 mesquite plants to the different treatments.

**FIGURE 6:**
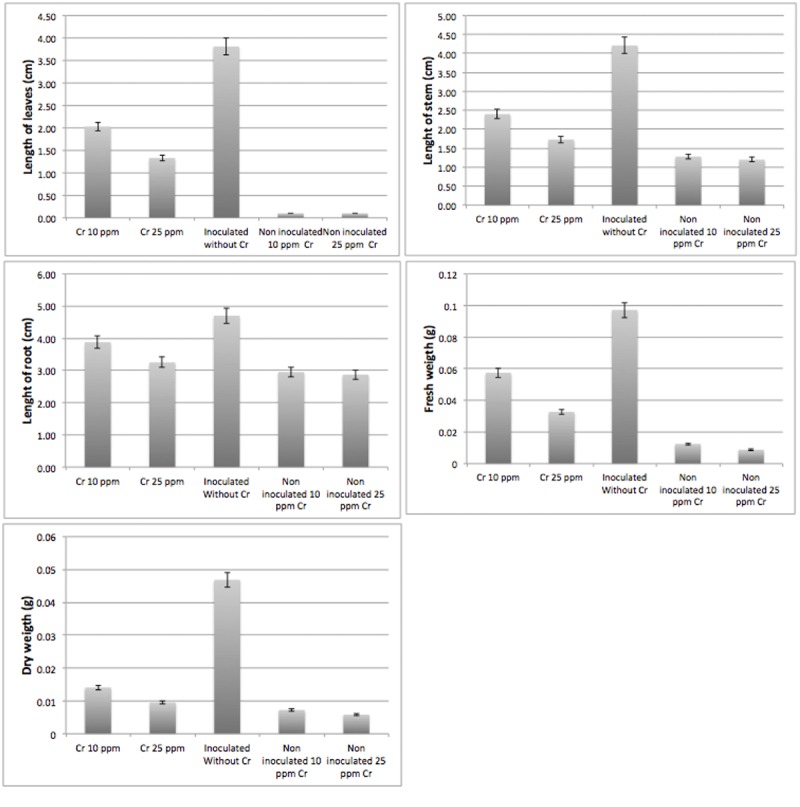
Plant growth parameters of seedlings inoculated with *Bacillus* sp. MH778713, with 0, 10 and 25 mg/L of Cr(VI) in Jensen’s free nitrogen medium. Non-inoculated plants grown in chromium-containing media were used as negative controls. Bars represent an average of twelve plants which were measured and weighed after completing 2 months of treatment.

Nodulating bacteria are known for their nitrogen-fixing capabilities. Since *Bacillus* sp. MH778713 was isolated from *Prosopis* nodules, its nitrogen-fixing capability was tested *in vivo* by growing twenty sprouting seeds of *Prosopis laevigata* in Jensen’s nitrogen-free medium; besides, the ability of *Bacillus* sp. MH778713 to assist plant-growth in presence of 0, 10 and 25 mg/L Cr (VI). Higher chromium concentrations were not allowed due to strong phytotoxic effects of this metal on the *Prosopis* seedlings. A representative single-plant picture of the twenty plants experiment is shown in Figure 5. Figures 5A,B showed that *Prosopis laevigata* itself was unable to grow in Jensen’s nitrogen-free medium, but *Prosopis* plants inoculated with *Bacillus* sp. MH778713 grew and developed true leaves, an indication of the nitrogen-fixing trait of *Bacillus* sp. MH778713. Furthermore, *Bacillus* sp. MH778713 assisted *Prosopis* to grow in the presence of 10 and 25 mg/L Cr (VI) (Figures 5C,D); uninoculated plants were unable to grow in the presence of 10 or 25 mg/L Cr and did not produce true leaves. Six plants of the experiment described previously were used to measuring plant growth parameters. The length of leaves and stems, and the weight of plants inoculated with *Bacillus* sp. MH778713 subjected to the chromium stress of 10 and 25 mg/L were significantly higher than those of uninoculated plants (Figure 6). Cr (VI) concentrations above 0.1 mg/kg in the soil are described as toxic for most of plants and some plants can accumulate up to 0.006–18 mg/kg of this metal (Shanker et al., 2005).

## Discussion

WXRF analysis indicated that explored soils contain considerable heavy metal concentrations e.g., aluminum, iron, titanium, zinc, copper, and chromium (Table 1). Aluminum is considered a toxic heavy metal which can be found in nature at concentrations of 4.5 × 10^3^–1 × 10^5^ mg/kg. High Al concentrations in the soil cause surface rooting in plants, changes in cell division, nutrition and chlorosis (Nilsson and Bergkvist, 1983; Taylor et al., 1998). The normal content of Al in plants is 1 × 10^3^ mg/kg (Clark, 1977; Foy et al., 1978; Slaski, 1994). In this study, *P. laevigata* trees accumulated up to 3.8 × 10^3^ mg/kg of Al in leaves and 4.1 × 10^4^ mg/kg in roots (Table 1), an indication of its bioaccumulation capability. In Table 1, Al accumulation was not shown by stem but shown by the root and leaf. This discrepancy could be due to differences in transpiration rate of shoot organs that affect Al distribution (Shen and Ma, 2001). Furthermore, the high oxalate concentrations in roots and leaves chelate Al forming a stable, non-phytotoxic complex of Al-oxalate that can be accumulated in those organs (Shen and Ma, 2001). When Al-oxalate is translocated from roots to leaves, Al-oxalate is converted to Al-citrate complex to be transported by xylem whose low concentrations are probably undetectable for our analytical methods.

Iron is the main component of the lithosphere (Kabata-Pendias, 2010). Typical amounts of Fe in the soil are 30–550 mg/kg; in acid soils values reach up to 2000 mg/kg (Wuana and Okieimen, 2011). The Fe concentration found in different species of plants is 220–1200 mg/kg (Price et al., 1972; Boardman, 1975; Markert, 1987). We found 51300 mg/kg in rhizospheric soil, and 9000 mg/kg in roots, 4800 mg/kg in leaves, and 3200 mg/kg in the stem of *Prosopis laevigata* exceeding by far the concentrations found in other plants. Titanium concentrations of soils are 1000–9000 mg/kg (Bain, 1976; Kiser et al., 2009). Ti participates in the nitrogen fixation process carry out by *Anabaena variabilis* (Cherchi and Gu, 2010) and stimulates the growth of some Rhizobia (Simon et al., 1990). Typical concentrations of Ti in plants are 0.15 and 80 mg/kg. High Ti concentrations produce chlorosis and necrotic spots on leaves (Wallace et al., 1977). The Ti concentration in our soil (5300 mg/kg) was into the average range reported but the concentration of Ti accumulated in the sheath of adult trees was two orders higher than those previously reported; demonstrating that *P. laevigata* trees are good Ti hyperaccumulator. Zinc typical concentrations in plants are 10–20 mg/kg. *Solanum nigrum* can accumulate up to 638 mg/kg of Zn in roots, 411 mg/kg in stems and 1054 mg/kg in leaves (Marques et al., 2006). *P. laevigata* plants also surpassed those bioaccumulation concentrations, showing that *P. laevigata* can tolerate higher levels of Zinc.

Typical chromium concentrations in soils are 5–120 mg/kg (Kabata-Pendias, 2010). High chromium content in soils causes poor crop growth (Wedepohl, 1974). In most of plants, chromium concentrations are 0.02–0.2 mg/kg. Laboratory experiments of *Prosopis* seedlings in association with *Glomus deserticola* grown in 80 or 160 mg Cr/kg soil, showed that Cr (III) and Cr (VI) are accumulated in roots and stem up to 700 or 1000 mg/kg (Arias et al., 2010, 2011). Although chromium concentration in our soil samples was high (435 and 208 mg/kg), plant tissues of *Prosopis* trees did not contain any detectable amount of chromium, which was unexpected. To investigate if endophytic microorganism of *Prosopis* could play any role in the detoxification of soil allowing *Prosopis* trees to grow, the diversity of associated bacteria was investigated.

From the 88 nodule-isolate strains, six of them resulted in high-Cr tolerance (10000–15000 mg/L), being the MH778713 strain the most tolerant. Among hyper Cr-tolerant microorganism known up to now are Streptomyces sp. CG52 (500 mg/L of Cr), *Staphylococcus aureus* and *Pediococcus pentosaceus* (2000 mg/L) (Srinath et al., 2002; Quintelas et al., 2008). Compared to *P. pentosaceus*, our MH778713 isolated tolerate 7.5-fold more Cr. All six Cr-tolerant strains of this study belonged to the genus *Bacillus*. Although *Bacillus* has not been described as legume symbiont, several *Bacillus* strains have been identified as endophytic bacteria. They have been isolated from a variety of plants, including legume plants as *Medicago polymorPha* (Chinnaswamy et al., 2018), *Dalbergia odorifera* (Lu et al., 2017), *Glycine max* (Hong et al., 2007), *Cajanus cajan* (Rajendran et al., 2007)*, Hedysarum spinosissimum* (Sbabou et al., 2016) and *Prosopis* species. Bacteria belonging to genus *Bacillus* have been isolated from the endosphere and rhizosphere of *Prosopis juliflora* (Khan et al., 2014; Abdelmoteleb et al., 2017) and *Prosopis farcta* nodules (Fterich et al., 2011). Though our *Bacillus* strains failed to induce nodule formation, the co-existence of *Bacillus* genus with rhizobial bacteria into legume nodule and the ability of *Bacillus* to influence the growth and fitness of plants has been well established (Arias et al., 2011). Thus, we reasoned, if Cr-tolerant strains assisted *Prosopis* trees to tolerate chromium toxicity, those strains might uptake chromium from the environment in addition to tolerate it.

Thus, chromium biosorption assays were carried out using the most Cr-tolerant strain, the *Bacillus* sp. MH778713. Results showed the removal of 116 mg/g of biomass when cells were exposed to 1474 mg/L of Cr (VI). *Bacillus circulans* and *Bacillus megaterium* can bioaccumulate 34.5 and 32.0 mg of chromium (VI)/g of dried weight (Cánovas et al., 2003). *B. coagulans* can absorb up to 1.5, 1.98 and 5.3 mg/g of biomass, in experiments with initial Cr concentrations of 10, 50 and100 mg/L (Srinath et al., 2002). A mixed culture of *Pseudomonas aeruginosa* and *Bacillus subtilis* have shown to uptake up to 1.44 mg Cr (VI)/g of biomass in aqueous solution (10 mg/L) (Quintelas et al., 2008). *Bacillus subtilis* can remove 63 mg/g of biomass in a solution of 100 mg/L after 6 h of exposure (Tarangini et al., 2009). Comparing with other *Bacillus* species, our *Bacillus* sp. MH778713 showed superior efficiency in the biosorption of chromium (VI), highlighting the potential of this strain for bioremediation of Cr contaminated sites.

The ability of genus *Bacillus* to produce compounds associated with an increase in size and weight in plants is well documented; for example, the AIA production (indolyl-3-acetic acid) (Sethuraman and Balasubramanian, 2010). *Bacillus* sp. MH778713, the most tolerant strain to chromium (VI), was not responsible for inducing nodulation in mesquite plants, but probably is a natural endophyte bacterium. This strain may produce substances that promote plant development, or may has systems to neutralize the negative effect of chromium (VI) such as Cr (VI) efflux pumps, Cr (VI) reduction to Cr (III), activations of enzymes involved in the Reactive Oxygen Species (ROS) detoxifying process, and repair DNA lesions (Bitas et al., 2013; Viti et al., 2014); all these mechanisms probably involved in assistance of plants to Cr adaptation. The results of *in vitro* inoculation of *Bacillus* sp. MH778713 on mesquite plants showed that this strain is capable of conferring tolerance to heavy metal (Cr) toxicity, since non-inoculated plants, exposed to high concentrations of chromium, did not develop true leaves (Figure 5) and showed smaller plant growth parameters (Figure 6).

## Conclusion

In the present study, *Prosopis laevigata*, an indigenous desert plant, showed the ability to hyperaccumulate heavy metals such as Al, Fe, Zn, Ti, and Zr in association with bacteria belonging to genus *Bacillus*. The *Bacillus* strain MH778713 isolated from *Prosopis* nodules showed high tolerance to chromium (VI), more than any other microorganism reported until now. Compared with other *Bacillus* species, *Bacillus* strain MH778713 showed a higher capacity to remove chromium (VI) from aqueous media, which could be beneficial for plants growing in soils with elevated chromium content. Further analyses will be needed to explore the full potential of *Bacillus* sp. MH778713 as an inoculant for plants in sustainable rehabilitation strategies of contaminated or disturbed land; as well as the nature of the substances and mechanisms involved in this tolerance.

## Data Availability

The datasets generated for this study can be found in GenBank/EMBL/DDBJ databases, MH778712/MH778713.

## Author Contributions

VR, AB, JM, and JAM conceived, designed, and directed the project, contributed to the interpretation of the results, and designed the figures. VR worked out almost all of the technical details, contributed to sample preparation, carried out the experiments, and performed the numerical calculations for the suggested experiments. VR, PL, and JC participated in the acquisition of the data. RB, MV, RC, JM-R, and LF supervised the work. JC carried out the taxonomic identification of plants. VR and PL manufactured the samples and performed the spectrometry measurements. AB and JM verified the analytical methods and analyzed the data. AB and JAM took the lead in the writing of the manuscript. All authors provided critical feedback, helped to shape the research, analysis, and manuscript, discussed the results, and contributed to the final version of the manuscript.

## Conflict of Interest Statement

The authors declare that the research was conducted in the absence of any commercial or financial relationships that could be construed as a potential conflict of interest.

## References

[B1] Abdelmoteleb A. Troncoso R. Gonzalez T. González D. (2017). Antifungical activity of autochthonous *bacillus subtilis* isolated from *prosopis juliflora* against phytopathogenic fungi. *Mycobiology* 45 385–391. 10.5941/MYCO.2017.45.4.385 29371807 PMC5780371

[B2] Aldrich M. V. Gardea-Torresdey J. L. Peralta-Videa J. R. Parsons J. G. (2003). Uptake and reduction of Cr (VI) to Cr (III) by mesquite (*Prosopis* spp.): chromate–plant interaction in hydroponics and solid media studied using XAS. *Environ. Sci. Technol.* 37 1859–1864. 10.1021/es0208916 12775058

[B3] Aldrich M. V. Peralta-Videa J. R. Parsons J. G. Gardea-Torresdey J. L. (2007). Examination of arsenic (III) and (V) uptake by the desert plant species mesquite (*Prosopis* spp.) using X-ray absorption spectroscopy. *Sci. Total Environ.* 379 249–255. 10.1016/j.scitotenv.2006.08.053 17055035

[B4] Álvarez E. Marcos M. L. Vaamonde C. Fernández-Sanjurjo M. J. (2003). Heavy metals in the dump of an abandoned mine in Galicia (NW Spain) and in the spontaneously occurring vegetation. *Sci. Total. Environ.* 313 185–197. 10.1016/s0048-9697(03)00261-4 12922070

[B5] Arias A. Peralta-Videa J. R. Ellzey J. T. Viveros M. N. Gardea-Torresdey J. L. (2010). Effects of *Glomus desertícola* inoculation on *Prosopis*: enhancing chromium and lead uptake and translocation as confirmed by x-ray mapping. *ICP-OES TEM Techniques. Environ. Exp. Bot.* 68 139–148. 10.1016/j.envexpbot.2009.08.009

[B6] Arias A. Peralta-Videa J. R. Ellzey J. T. Viveros M. N. Ren M. Mokgalaka N. S. (2011). Plant growth and metal distribution in tissues of *Prosopis juliflora-velutina* grown on chromium-contaminated soil in the presence of *Glomus desertícola*. *Environ. Sci. Technol.* 44 7272–7279. 10.1021/es1008664 20795657 PMC4337994

[B7] Ashraf M. A. Hussain I. Rasheed R. Iqbal M. Riaz M. Arif M. S. (2017). Advances in microbe-assisted reclamation of heavy metal contaminated soils over the last decade: a review. *J. Environ. Manag.* 198 132–143. 10.1016/j.jenvman.2017.04.060 28456029

[B8] Bain D. C. (1976). A titanium-rich soil clay. *Eur. J. Soil. Sci.* 27 68–70. 10.1111/j.1365-2389.1976.tb01976.x

[B9] Bankar A. Ameeta R. Kumar R. Smita S. Zinjarde S. (2009). Removal of chromium (VI) ions from aqueous solution by adsorption onto two marine isolates of *Yarrowia lipolytica*. *J. Hazard. Mater.* 170 487–494. 10.1016/j.jhazmat.2009.04.070 19467781

[B10] Bitas V. Kim H. Bennett J. W. Kang S. (2013). Sniffing on microbes: diverse roles of microbial volatile organic compounds in plant health. *Mol. Plant Microbe Interac.* 268 835–843. 10.1094/MPMI-10-12-0249-CR 23581824

[B11] Boardman N. K. (1975). “Trace elements in photosynthesis,” in *Trace Elements in Soil-Plant-Animal Systems*, eds Nicholas P. J. D. Egan A. R. (New York., NY: Academic Press).

[B12] Campos V. Moraga R. Yánez J. Zaror C. Mondaca M. (2005). Chromate reduction by *Serratia marcescens* isolated from tannery effluent. *Bull. Environ. Cont. Toxic.* 75 400–406. 10.1007/s00128-005-0767-z 16222516

[B13] Cánovas D. Cases I. De Lorenzo V. (2003). Heavy metal tolerance and metal homeostasis in *Pseudomonas putida* as revealed by complete genome analysis. *Environ. Microbiol.* 5 1242–1256. 10.1111/j.1462-2920.2003.00463.x 14641571

[B14] Cappa J. J. Pilon-Smits E. A. H. (2014). Evolutionary aspects of hyperaccumulation. *Plant* 239 267–275. 10.1007/s00425-013-1983-0 24463931

[B15] Chaney R. L. Malik M. Brown L. Brewer S. L. Angle J. S. Baker A. J. M. (1997). Phytoremediation of soil metals. *Curr. Opin. Biotechnol.* 8 279–284. 9206007 10.1016/s0958-1669(97)80004-3

[B16] Cherchi C. Gu Z. (2010). Impact of titanium dioxide nanomaterials on nitrogen fixation rate and intracellular nitrogen storage in *Anabaena variabilis*. *Environ. Sci. Technol.* 44 8302–8307. 10.1021/es101658p 20853867

[B17] Chinnaswamy A. Coba de la Peña T. Stoll A. de la Peña Rojo D. Bravo J. Rincón A. (2018). A nodule endophytic *Bacillus megaterium* strain isolated from *Medicago polymorpha* enhances growth, promotes nodulation by *Ensifer medicae* and alleviates salt stress in alfalfa plants. *Ann. Appl. Biol.* 172 295–308. 10.1111/aab.12420

[B18] Claret F. Tournassat C. Crouzet C. Gaucher E. Schäfer T. Braibant G. (2011). Metal speciation in landfill leachates with a focus on the influence of organic matter. *Waste Manag.* 31 2036–2045. 10.1016/j.wasman.2011.05.014 21705206

[B19] Clark R. B. (1977). Effect of aluminum on the growth and mineral elements of Al-tolerant and Al-intolerant corn. *Plant. Soil.* 47 653–659.

[B20] Corral A. Morales Y. E. Pazos L. A. Ramírez A. Martínez R. D. Muñoz J. (2012). Quantification of cultivable bacteria by the “massive stamping drop plate” method. *Rev. Colomb. Biotecnol.* 14 147–156.

[B21] Davies F. T. Puryear J. D. Newton R. J. Egilla J. N. Grossi J. A. S. (2002). Mycorrhizal fungi increase chromium uptake by sunflower plants: influence on tissue mineral concentration, growth, and gas exchange. *J. Plant Nutr.* 25 2389–2407. 10.1081/pln-120014702

[B22] Dimkpa C. Merten D. Svatoš A. Büchel G. Kothe E. (2009). Siderophores mediate reduced and increased uptake of cadmium by *Streptomyces tendae* F4 and sunflower (*Helianthus annuus*), respectively. *J. Appl. Microbiol.* 107 1687–1696. 10.1111/j.1365-2672.2009.04355.x 19457036

[B23] Elangovan R. Abhipsa S. Rohit B. Ligy P. Chandraraj K. (2006). Reduction of Cr (VI) by a *Bacillus* sp. *Biotechnol. Lett.* 28 247–252. 10.1007/s10529-005-5526-z 16555008

[B24] Ernst W. H. O. (1995). Sampling of plant material for chemical analysis. *Sci Total Environ.* 176 15–24. 10.1016/0048-9697(95)04826-X

[B25] Everts J. (1997). Ecotoxicology for risk assessment in arid zones: some key issues. *Arch. Environ. Contam. Toxicol.* 32 1–10. 10.1007/s002449900149 9002429

[B26] Fao Conservation Guide 20. (1989). *Arid zone forestry: A Guide for Field Technicians. ISBN 92-5-102809-5*. Rome: Food and Agriculture Organization.

[B27] Felker P. Kanell G. H. Clark P. R. (1983). Biomass production of *Prosopis* species (Mesquite), leucaena, and other leguminous trees grown under heat/drought stress. *Forest. Sci.* 29 592–606.

[B28] Foy C. D. Chaney R. L. White M. C. (1978). The physiology of metal toxicity in plants. *Annu. Rev. Physiol.* 29 511–519.

[B29] Francisco R. Alpoim M. C. Morais P. V. (2002). Diversity of chromium-resistantant reducing bacteria in a chromium-contaminated activated sludge. *J. Appl. Microbiol.* 92 837–843. 10.1046/j.1365-2672.2002.01591.x 11972686

[B30] Fterich A. Mahdhi M. Pajuelo E. Rivas R. Mars M. (2011). Characterization of root-nodulating bacteria associated to *Prosopis farcta* growing in the arid regions of Tunisia. *Arch. Microbiol.* 193 385–397. 10.1007/s00203-011-0683-z 21359955

[B31] Gardea-Torresdey J. L. de la Rosa G. Peralta-Videa P. (2004). Use of phytofiltration technologies in the removal of heavy metals: a review. *Pure*. *Appl. Chem.* 76 801–813. 10.1351/pac200476040801

[B32] Glass D. J. (1999). *United States and International Markets for Phytoremediation.* Needham: D. Glass Associates Inc.

[B33] Goulhen F. Gloter A. Guyot F. Bruschi M. (2006). Cr (VI) detoxification by *Desulfovibrio vulgaris* strain Hildenborough: microbe–metal interaction studies. *Appl. Microbiol. Biotechnol.* 71 892–897. 10.1007/s00253-005-0211-7 16896506

[B34] Gowd S. Govil P. K. (2018). Distribution of heavy metals in surface water of ranipet industrial area in Tamil Nadu. *Ind. Environ. Monit. Assess* 136 197–207. 10.1007/s10661-007-9675-5 17457685

[B35] Hong H. P. Zhang H. Olhoft P. Hill S. C. Wiley H. O. Toren E. C. (2007). *Organogenic callus* as the target for plant regeneration and transformation via *Agrobacterium* in soybean (*Glycine max* (L.) Merr.). In Vitro Cell. *Dev. Biol. Plant* 43 558–568. 10.1007/s11627-007-9066-1

[B36] Hou J. Liu W. Wang B. Wang Q. Luo Y. Franks A. E. (2015). PGPR enhanced phytoremediation of petroleum contaminated soil and rhizosphere microbial community response. *Chemosphere* 138 592–598. 10.1016/j.chemosphere.2015.07.025 26210024

[B37] Jayaram K. Prasad M. N. V. (2009). Removal of Pb (II) from aqueous solution by seed powder of *Prosopis juliflora* D.C. *J. Hazard. Mater.* 169 991–997. 10.1016/j.jhazmat.2009.04.048 19464107

[B38] Kabata-Pendias A. (2010). *Trace Elements in Soils and Plants*, 4th Edn Boca Raton,: CRC Press.

[B39] Kalbitz K. Wennrich R. (1998). Mobilization of heavy metals and arsenic in polluted wetland soils and its dependence on dissolved organic matter. *Sci. Total. Environ.* 209 27–39. 10.1016/s0048-9697(97)00302-1 9496662

[B40] Khan M. U. Sessitsch A. Harris M. Fatima K. Imran A. Arslan M. (2014). Cr-resistant rhizo- and endophytic bacteria associated with *Prosopis juliflora* and their potential as phytoremediation enhancing agents in metal-degraded soils. *Front. Plant. Sci.* 5:755. 10.3389/fpls.2014.00755 25610444 PMC4284999

[B41] Kiser M. A. Wesrehoff P. Benn T. Wang Y. Perez-Rivera J. Hristovski K. (2009). Titanium nanomaterial removal and release from wastewater treatment plants. *Environ. Sci. Technol.* 43 6757–6763. 10.1021/es901102n 19764246

[B42] Kotas J. Stasicka Z. (2000). Chromium occurrence in the environment and methods of this speciation. *Environ. Pollut.* 107 263–283. 10.1016/s0269-7491(99)00168-2 15092973

[B43] Kumar S. Stecher G. Tamura K. (2016). MEGA7: molecular evolutionary genetics analysis version 7.0 for bigger datasets. *Mol. Biol. Evol.* 33 1870–1874. 10.1093/molbev/msw054 27004904 PMC8210823

[B44] Lu J. Yang F. Wang S. Ma H. Liang J. Chen Y. (2017). Co-existence of rhizobia and diverse non-rhizobial bacteria in the rhizosphere and Nodules of *Dalbergia odorifera* seedlings inoculated with *Bradyrhizobium elkanii*, rhizobium multihospitium–like and burkholderia pyrrocinia–like strains. *Front. Microbiol.* 8:2255. 10.3389/fmicb.2017.02255 29209289 PMC5702347

[B45] Mahar M. Wang P. Ali A. Kumar M. Hussain A. Wang Q. (2016). Challenges and opportunities in the phytoremediation of heavy metals contaminated soils: a review. *Ecotoxicol. Environ. Saf.* 126 111–121. 10.1016/j.ecoenv.2015.12.023 26741880

[B46] Marguí E. Hidalgo M. Queralt I. (2007). XRF spectrometry for trace elements analysis of vegetation samples. *Spectrosc. Eur.* 19 3–10.

[B47] Markert B. (1987). Multielementanalytik: mögliche darstellungsweisen von messdaten. *Fresenius Z. Anal. Chem.* 315 327–329.

[B48] Marques A. F. Queralt I. Carvalho M. L. Bordalo M. (2003). Total reflection X-ray fluorescence and energy-dispersive X-ray fluorescence analysis of runoff water and vegetation from abandoned mining of Pb–Zn ores. *Spectrochimica Acta Part B: At. Spectrosc.* 58 2191–2198. 10.1016/s0584-8547(03)00213-1

[B49] Marques A. P. G. C. Oliveira R. S. Rangel A. O. S. S. Castro P. M. L. (2006). Zinc accumulation in solanum nigrum are enhanced by different arbuscular mycorrhizal fungi. *Chemosphere* 65 1256–1263. 10.1016/j.chemosphere.2006.03.022 16650459

[B50] Martínez-Pérez A. López P. A. Gil-Muñoz A. Cuevas-Sánchez J. A. (2012). Plantas silvestres útiles y prioritarias identificadas en la mixteca poblana. *México. Acta Bot. Mex.* 98 73–98.

[B51] McLean J. A. Acon B. W. Montaser A. Singh J. Pritchard D. E. Patierno S. R. (2000). Determination of chromium in human lung fibroblast cells using a large bore—direct injection high-efficiency nebulizer with inductively coupled plasma mass spectrometry. *Appl. Spectrosc.* 55 659–663. 10.1366/0003702001950120

[B52] Megharaj M. Avudainayagam S. Naidu R. (2003). Toxicity of hexavalent chromium and its reduction by bacteria isolated from soil contaminated with tannery waste. *Curr. Microbiol.* 47 51–54. 10.1007/s00284-002-3889-0 12783193

[B53] Mench M. Martin E. (1991). Mobilization of cadmium and other metals from two soils by root exudates of *Zea mays* L., *Nicotiana tabacum* L. and *Nicotiana rustica* L. *Plant. Soil* 132 187–196. 10.1007/BF00010399

[B54] Mokgalaka N. S. Flores E. Castillo H. Peralta-Videa J. R. Gardea-Torresdey J. L. (2008). Toxicity of Arsenic (III) and (V) on plant growth, element uptake, and total amylolytic activity of mesquite (*Prosopis juliflora* and P. velutina). Inter. *J. Phytorem.* 10 47–60. 10.1080/15226510701827069 18709931

[B55] Morales Y. Juárez D. Aragón C. Mascarua M. Bustillos R. Fuentes L. (2011). Growth response of maize plantlets inoculated with *Enterobacter* spp., as a model for alternative agriculture. *Rev. Argent. Microbiol.* 43 287–293. 10.1590/S0325-75412011000400009 22274827

[B56] Mulligan C. N. Yong R. N. Gibbs B. F. (2001). Heavy metal removal from sediments by biosurfactants. *J. Hazard. Mater.* 85 1–2. 11463506 10.1016/s0304-3894(01)00224-2

[B57] Nicholas K. B. Nicholas J. H. (1997). *GeneDoc: a tool for editing and annotating multiple sequence alignments.* Berlin: ScienceOpen, Inc.

[B58] Nilsson S. I. Bergkvist B. (1983). Aluminium chemistry and acidification processes in a shallow podzol on the Swedish westcoast. *Water. Air. Soil. Pollut.* 20 311–329.

[B59] Nivethitha P. Thangavel P. Prince W. S. P. M. Subburam V. (2002). Identification of heavy metal accumulating plants and their use in reclamation of soil contaminated with heavy metals. *Eco. Env. Cons.* 8 249–251.

[B60] Park J. H. Choppala G. K. Bolan N. S. Chung J. W. Chuasavathi T. (2011). Biochar reduces the bioavailability and phytotoxicity of heavy metals. *Plant Soil* 348 439–451. 10.1007/s11104-011-0948-y

[B61] Pérez G. Tamariz V. López L. Hernández F. Castelán R. García W. A. (2018). Atoyac river pollution in the metropolitan area of puebla. *México. Water.* 10 267–284. 28471407

[B62] Price C. A. Clark H. E. Funkhouser E. A. (1972). *Functions of Micronutrients in Plants. In Micronutrients in Agriculture.* Madison, Wisconsin: Soil Science Society of America, Inc., 231.

[B63] Quintelas C. Fernandes B. Castro J. Figueiredo H. Tavares T. (2008). Biosorption of Cr (VI) by a *Bacillus coagulans* biofilm supported on granular activated carbon (GAC). *Chem. Eng. J.* 136 195–203. 10.1016/j.cej.2007.03.082 17933461

[B64] Rajendran G. Mistry S. Desai A. J. (2007). Functional expression of *Escherichia coli* fhuA gene in Rhizobium spp. of Cajanus cajan provides growth advantage in presence of Fe3+: ferrichrome as iron source. *Arch. Microbiol.* 187 257–264. 10.1007/s00203-006-0191-8 17136381

[B65] Rajkumar M. Nagendran R. (2005). Characterization of a novel Cr6+ reducing *Pseudomonas* sp. with plant growth-promoting potential. *Curr. Microbiol.* 50 266–271. 10.1007/s00284-005-4470-4 15886910

[B66] Rehman A. Zahoor A. Muneer B. Hasnain B. (2008). Chromium tolerance and reduction potential of a *Bacillus* sp. ev3 isolated from metal contaminated wastewater. *Bull. Environ. Contam. Toxicol.* 81 25–29. 10.1007/s00128-008-9442-5 18498008

[B67] Sajjad A. Saeed S. Bashir M. A. (2012). Spatial variation in pollinator communities and productive performance of *Prosopis juliflora* (Fabaceae). *J. Poll. Ecol.* 8 59–66.

[B68] Sbabou L. Idir Y. Bruneel O. Le Queré A. Aurag J. (2016). Characterization of root-nodule bacteria isolated from *hedysarum spinosissimum* l, growing in mining sites of northeastern region of morocco. . *SOJ. Microbiol Infect. Dis.* 4 1–8. 10.15226/sojmid/4/3/00156

[B69] Schaeffer A. B. Fulton M. (1933). A simplified method of staining endospores. *Science* 77:194. 10.1126/science.77.1990.194 17741261

[B70] Schell D. Beermann C. (2014). Fluidized bed microencapsulation of *Lactobacillus reuteri* with sweet whey and shellac for improved acid resistance and in-vitro gastro-intestinal survival. *Food. Res. Int.* 62 308–314. 10.1016/j.foodres.2014.03.016

[B71] Senthilkumar P. Prince W. S. Sivakumar S. Subbhuraam C. V. (2005). *Prosopis juliflora*- a green solution to decontaminate heavy metal (Cu and Cd) contaminated soils. *Chemosphere* 60 1493–1496. 10.1016/j.chemosphere.2005.02.022 16054919

[B72] Sethuraman P. Balasubramanian N. (2010). Removal of Cr (VI) from aqueous solution using *Bacillus subtilis*, *Pseudomonas aeruginosa* and *Enterobacter cloacae*. *Int. J. Eng. Sci.* 2 1811–1825. 10.3390/ma8125461 28793717 PMC5458844

[B73] Shanker A. K. Cervantes C. Loza-Tavera H. Avudainayagam S. (2005). Chromium toxicity in plants. *Environ. Int.* 31 739–753. 10.1016/j.envint.2005.02.003 15878200

[B74] Shen R. F. Ma J. F. (2001). Distribution and mobility of aluminium in an Al-accumulating plant. *Fagopyrum esculentum. Moench J. Exp. Bot.* 52 1683–1687. 10.1093/jexbot/52.361.1683 11479333

[B75] Simon L. Balazsy S. Balogh A. Pais I. (1990). Effect of titanium on the growth of *Bradyrhizobium japonicum* and *Bradyrhizobium lupini* strains. *Acta. Microbiol. Pol.* 39 51–57.

[B76] Slaski J. J. (1994). Differences in the metabolic response of root tips of wheat and rye to aluminium stress. *Plant Soil* 167 165–171. 10.1007/bf01587612

[B77] Somasegaran P. Hoben H. J. (1994). *Handbook for Rhizobia: Methods in Legume-Rhizobium technology.* Berlin: Springer-Verlag.

[B78] Srinath T. Verma T. Ramteke P. W. Garg S. K. (2002). Chromium (VI) biosorption and bioaccumulation by chromate resistant bacteria. *Sci. Env. Technol. Chemosphere* 48 427–435. 10.1016/s0045-6535(02)00089-9 12152745

[B79] Tamura K. Peterson D. Peterson N. Stecher G. Nei M. Kumar S. (2011). MEGA5: molecular evolutionary genetics analysis using maximum likelihood, evolutionary distance, and maximum parsimony methods. *Mol. Biol Evol.* 28 2731–2739. 10.1093/molbev/msr121 21546353 PMC3203626

[B80] Tarangini K. Kumar A. Satpathy G. R. Sangal V. K. (2009). Statistical optimization of process parameters for cr (vi) biosorption onto mixed cultures of *Pseudomonas aeruginosa* and *Bacillus subtilis*. *Clean–Soil Air.Water* 37 319–327. 10.1002/clen.20090033

[B81] Taylor G. J. Blarney F. P. Edwards D. G. (1998). Antagonistic and synergistic interactions between aluminum and manganese on growth of *Vigna unguiculata* at low ionic strength. *Physiol. Plant* 104 183–194. 10.1034/j.1399-3054.1998.1040206.x

[B82] Thacker U. Madamwar D. (2005). Reduction of toxic chromium and partial localization of chromium reductase activity in bacterial isolate DM1. *World. J. Microbiol. Biotechnol.* 21 891–899. 10.1007/s11274-004-6557-7

[B83] Thompson J. D. (1997). The CLUSTAL_X windows interface: flexible strategies for multiple sequence alignment aided by quality analysis tools. *Nucleic. Acids. Res.* 25 4876–4882. 10.1093/nar/25.24.4876 9396791 PMC147148

[B84] Ullah A. Heng S. Farooq M. Fahad S. Yang X. (2015). Phytoremediation of heavy metals assisted by plant growth promoting (PGP) bacteria: a review. *Environ. Exp. Bot.* 117 28–40. 10.1016/j.envexpbot.2015.05.001

[B85] Viamajala S. Smith W. Sani R. K. Apel W. A. Petersen J. N. Neal A. L. (2007). Isolation and characterization of Cr (VI) reducing *Cellulomonas* spp. *from subsurface soils: implications for long-term chromate reduction*. *Bioresour. Technol.* 98 612–622. 10.1016/j.biortech.2006.02.023 16644211

[B86] Vincent J. M. (1970). *A Manual for the Practical Study of Root-Nodule Bacteria.* Oxford: Blackwell.

[B87] Viti C. Marchi E. Decorosi F. Giovannetti L. (2014). Molecular mechanisms of Cr (VI) resistance in bacteria and fungi. *FEMS Microbiol. Rev.* 38 633–659. 10.1111/1574-6976.12051 24188101

[B88] Wallace A. Alexander G. V. Chaudhry F. M. (1977). Phytotoxicity of cobalt, vanadium, titanium, silver and chromium. *Commun. Soil. Sci. Plant. Anal.* 8 751–756. 10.1080/00103627709366769

[B89] Wang L. Ji B. Hu Y. Liu R. Sun W. (2017). A review on in situ phytoremediation of mine tailings. *Chemosphere.* 184 594–600. 10.1016/j.chemosphere.2017.06.025 28623832

[B90] Wedepohl K. H. (ed.) (1974). *Handbook of Geochemistry.* Berlin: Springer-Verlag.

[B91] Wuana R. A. Okieimen F. E. (2011). Heavy metals in contaminated soils: a review of sources, chemistry, risks and best available strategies for remediation. *Commun. Soil. Sci. Plant. Anal.* 42 111–122.

[B92] Xun F. Xie B. Lui S. Guo C. (2015). Effect of plant growth-promoting bacteria (PGPR) and arbuscular mycorrhizal fungi (AMF) inoculation on oats in saline-alkali soil contaminated by petroleum to enhance phytoremediation. *Environ. Sci. Pollut. Res. Int.* 22 598–608. 10.1007/s11356-014-3396-4 25091168

[B93] Young H. K. Dong S. L. Kim H. B. (2003). *Vibrio harveyi* is also a chromate reductase. *Appl. Environ. Microbiol.* 69 4390–4395. 10.1128/aem.69.8.4390-4395.2003 12902220 PMC169119

[B94] Zappala M. N. Ellzey J. T. Bader J. Peralta-Videa J. R. Gardea-Torresdey J. L. (2014). Effects of copper sulfate on seedlings of *Prosopis pubescens* (Screwbean Mesquite). *Inter. J. Phytorem.* 16 1031–1041. 10.1080/15226514.2013.810582 24933900 PMC4061504

