# Supplementary material for: Chromium Hyper-Tolerant Bacillus sp. MH778713 Assists Phytoremediation of Heavy Metals by Mesquite Trees (Prosopis laevigata)
Source: Front Microbiol. 2019 Aug 13;10:1833. doi: 10.3389/fmicb.2019.01833 (PMC6700308; doi:10.3389/fmicb.2019.01833)
Supplement: Supplementary file 1 [file Data_Sheet_1.pdf]

Chromium hyper-tolerant Bacillus sp. assists phytoremediation of heavy metals by mesquite trees (Prosopis laevigata)

Verónica Ramírez, Antonino Baez, Primavera López, Rocío Bustillos, Migue-Ángel Villalobos, Ricardo Carreño, José-Luis Contreras, Jesús Muñoz-Rojas, Luis Ernesto Fuentes, Javier Martínez, José-Antonio Munive

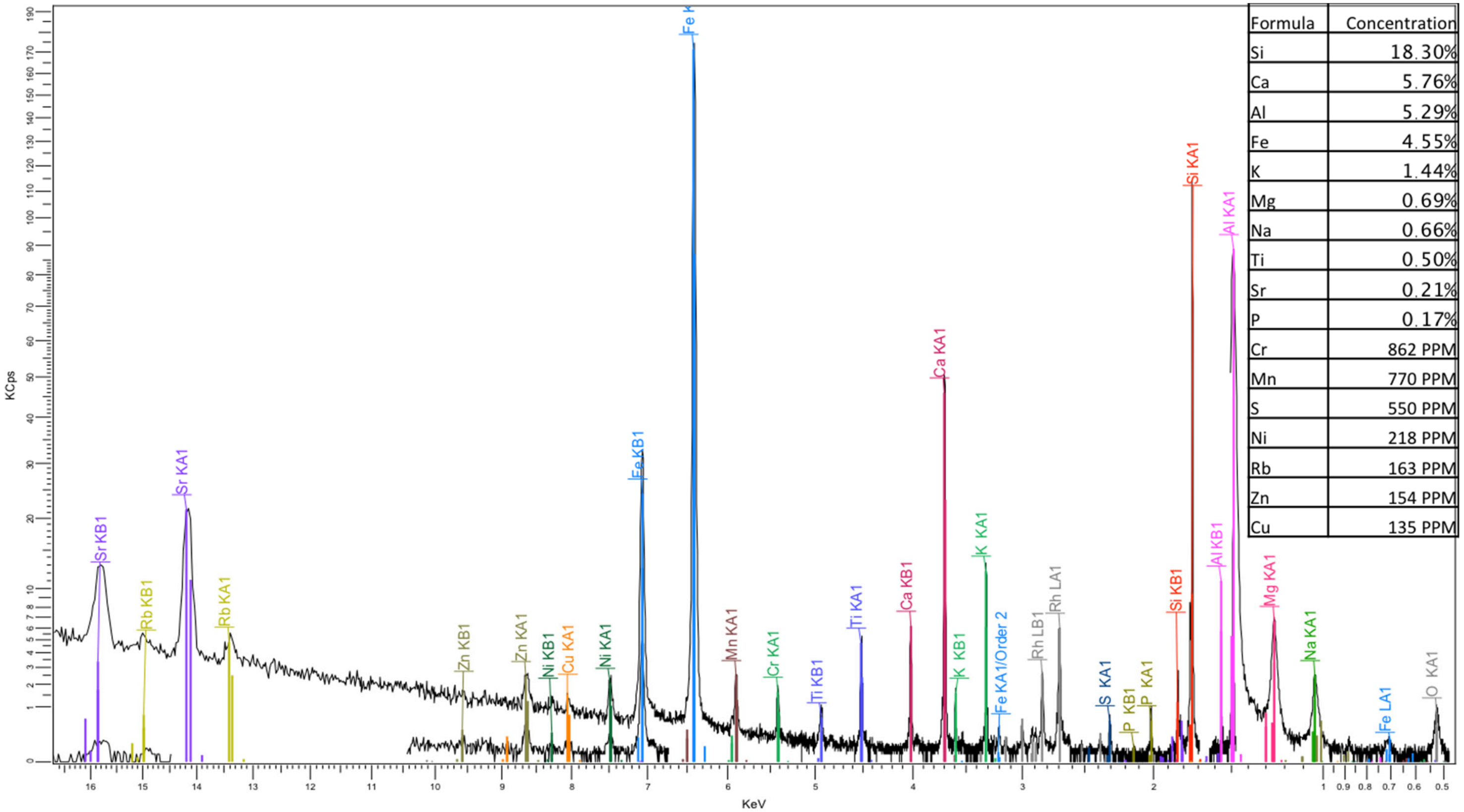

Formula	Concentration
Si	18.30%
Ca	5.76%
Al	5.29%
Fe	4.55%
K	1.44%
Mg	0.69%
Na	0.66%
Ti	0.50%
Sr	0.21%
P	0.17%
Cr	862 PPM
Mn	770 PPM
S	550 PPM
Ni	218 PPM
Rb	163 PPM
Zn	154 PPM
Cu	135 PPM

Fig S1. WDXRF (Wavelength Dispersive X-Ray Fluorescence) spectrum showing heavy metal content in soil sediment from Nexapa River region.

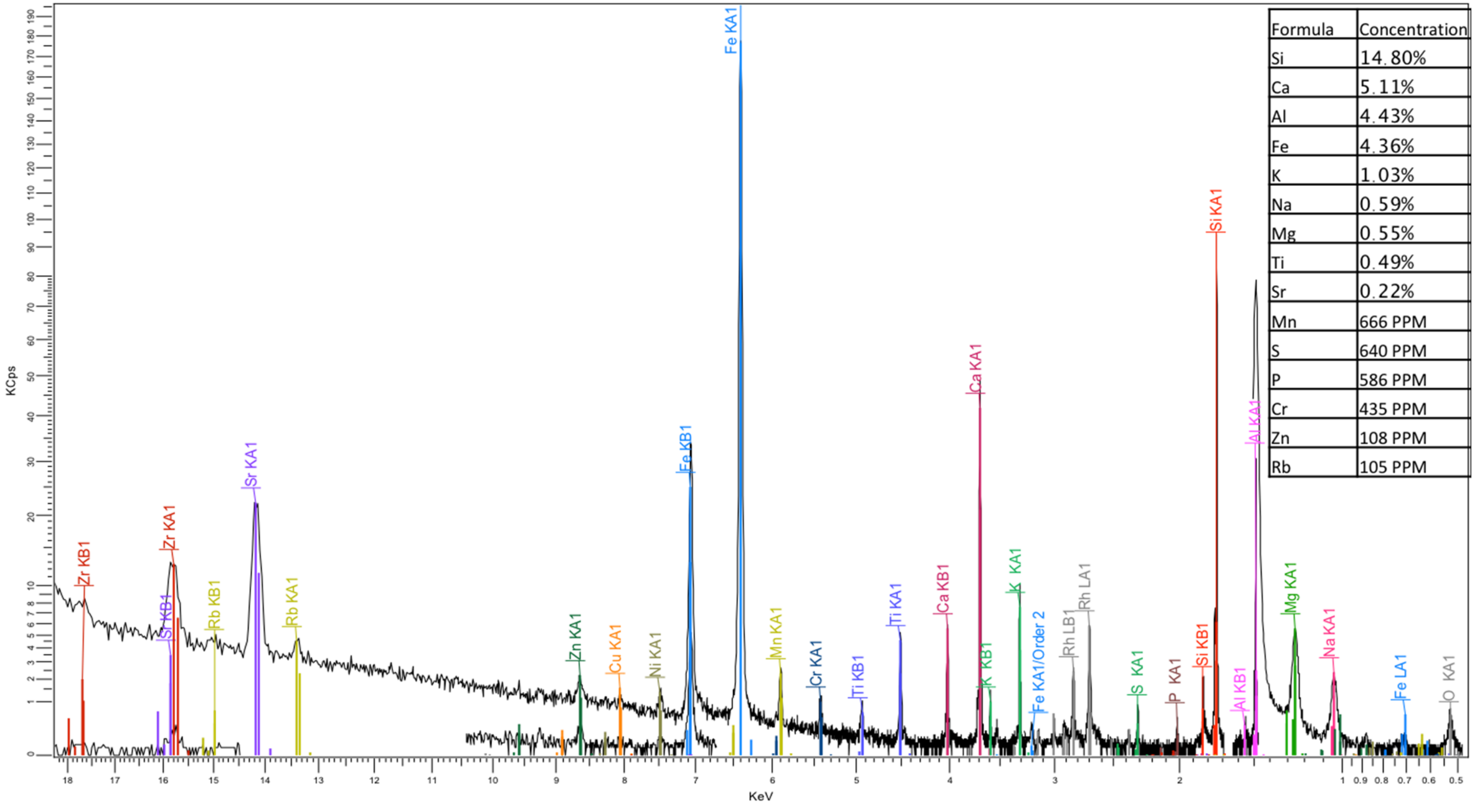

Formula	Concentration
Si	14.80%
Ca	5.11%
Al	4.43%
Fe	4.36%
K	1.03%
Na	0.59%
Mg	0.55%
Ti	0.49%
Sr	0.22%
Mn	666 PPM
S	640 PPM
P	586 PPM
Cr	435 PPM
Zn	108 PPM
Rb	105 PPM

Fig S2. WDXRF (Wavelength Dispersive X-Ray Fluorescence) spectrum showing heavy metal content in non-rhizospheric soil from Nexapa River region.

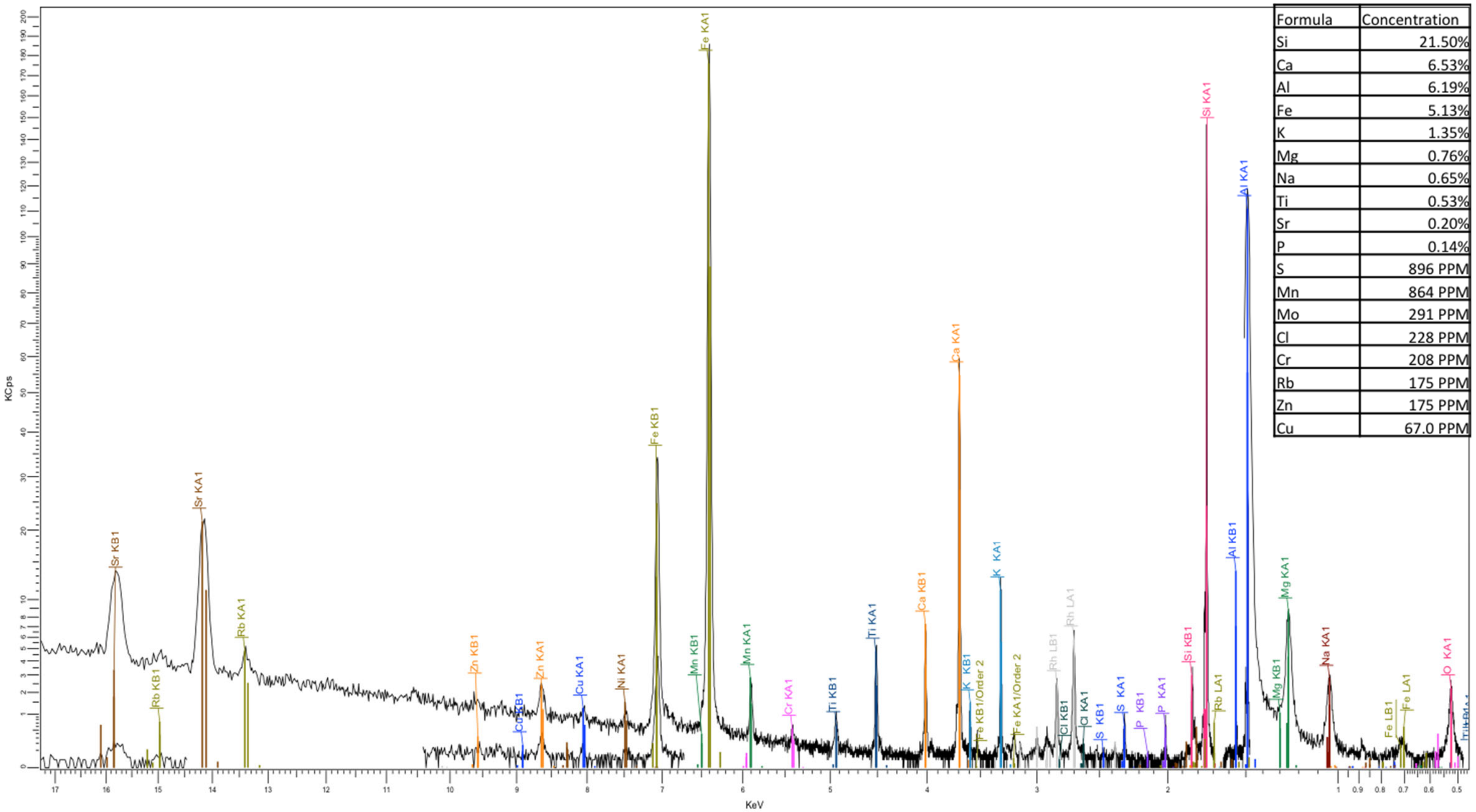

Formula	Concentration
Si	21.50%
Ca	6.53%
Al	6.19%
Fe	5.13%
K	1.35%
Mg	0.76%
Na	0.65%
Ti	0.53%
Sr	0.20%
P	0.14%
S	896 PPM
Mn	864 PPM
Mo	291 PPM
Cl	228 PPM
Cr	208 PPM
Rb	175 PPM
Zn	175 PPM
Cu	67.0 PPM

Fig S3. WDXRF (Wavelength Dispersive X-Ray Fluorescence) spectrum showing heavy metal content in rhizospheric soil from Nexapa River region.

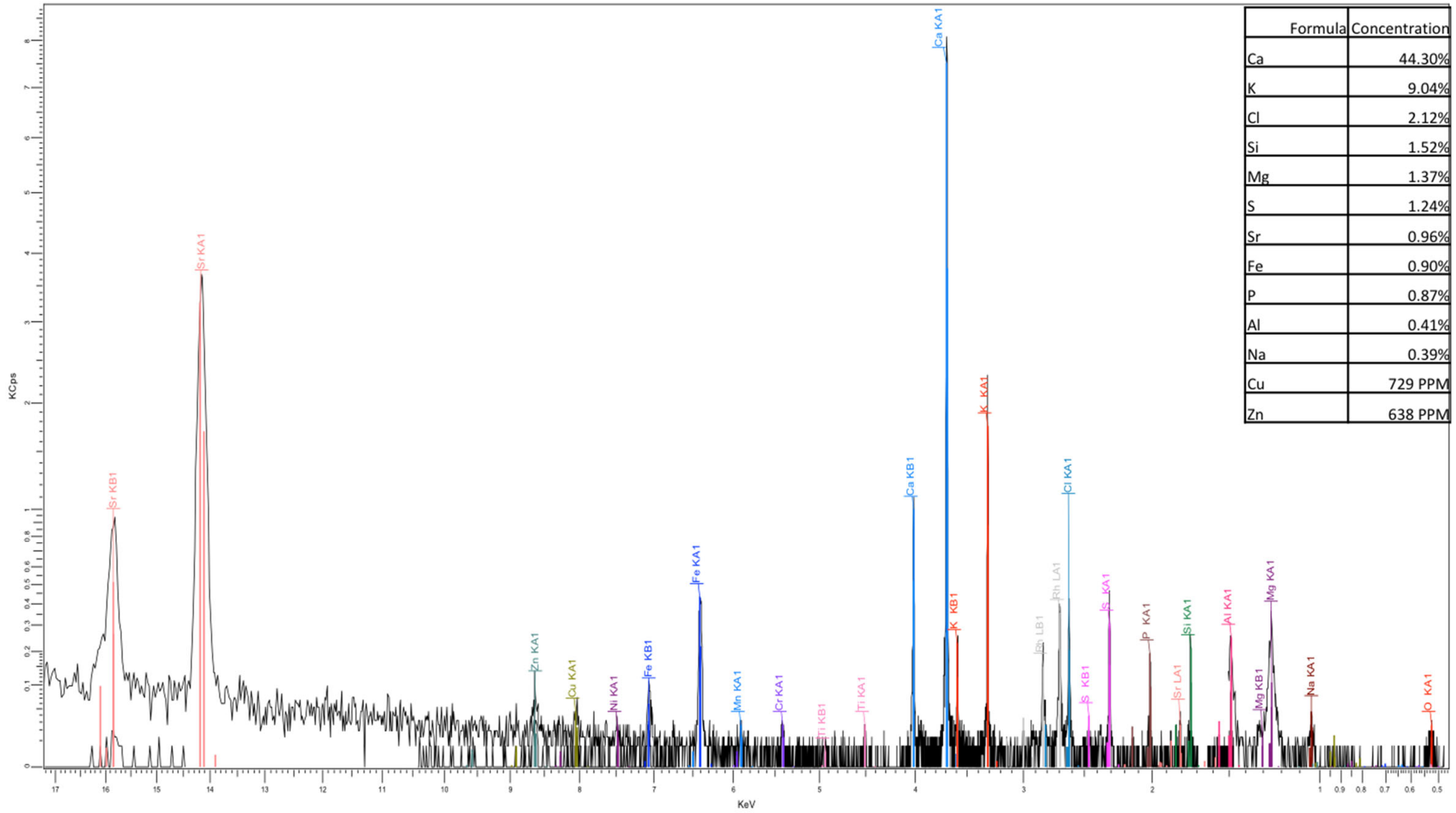

Fig S4. WDXRF (Wavelength Dispersive X-Ray Fluorescence) spectrum showing heavy metal content in roots of *Prosopis laevigata* plants from Nexapa River region.

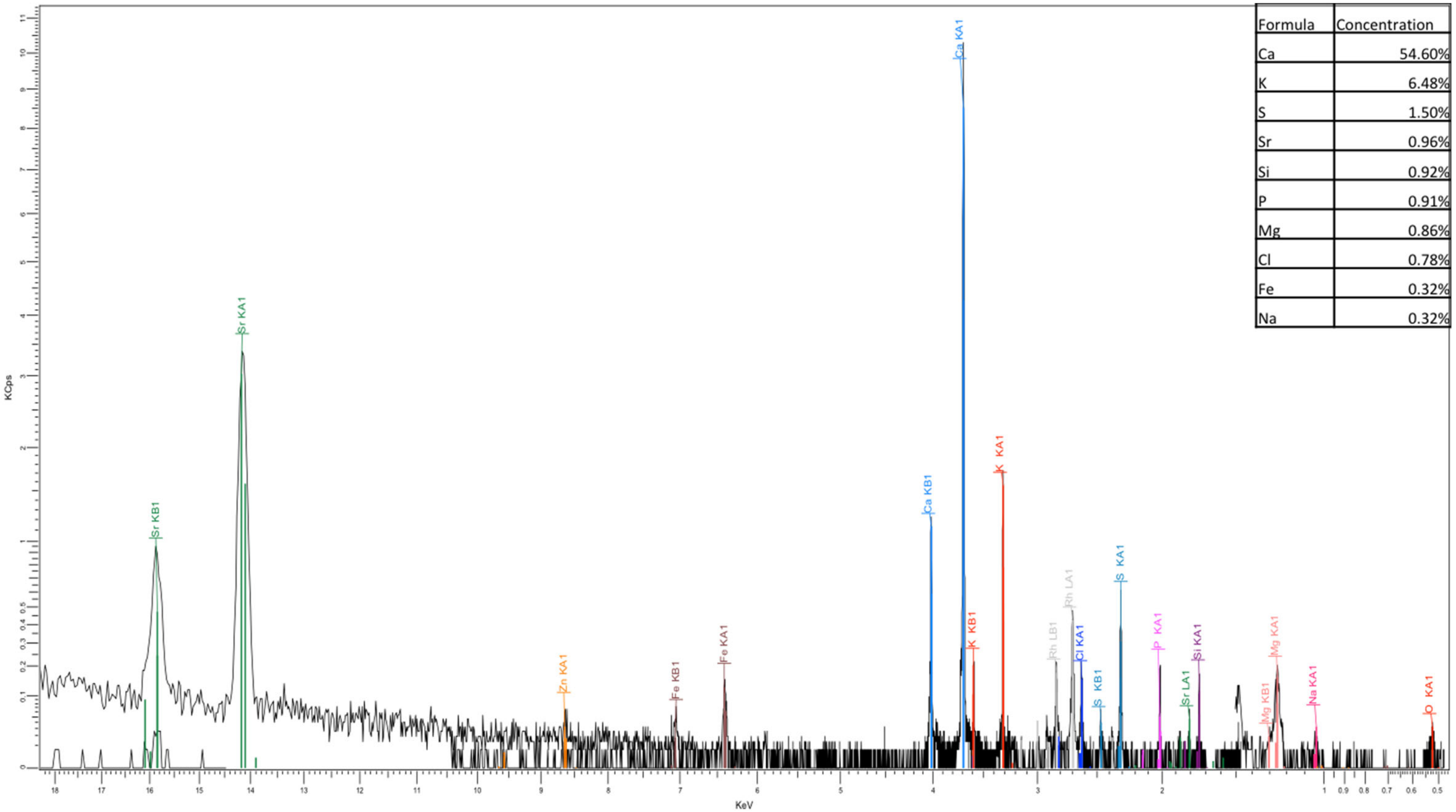

Formula	Concentration
Ca	54.60%
K	6.48%
S	1.50%
Sr	0.96%
Si	0.92%
P	0.91%
Mg	0.86%
Cl	0.78%
Fe	0.32%
Na	0.32%

Fig S5. WDXRF (Wavelength Dispersive X-Ray Fluorescence) spectrum showing heavy metal content in stems of *Prosopis laevigata* plants from Nexapa River region.

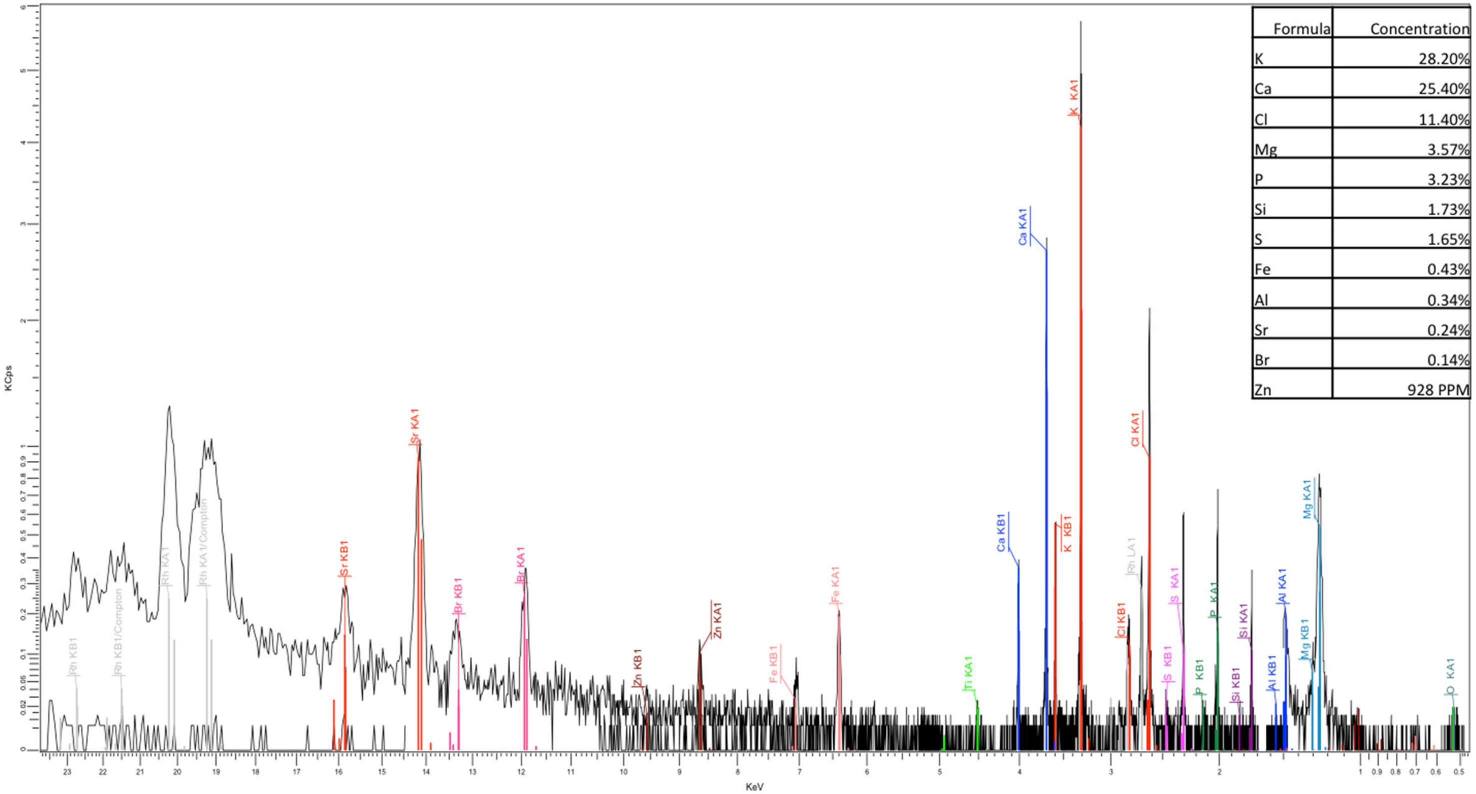

Formula	Concentration
K	28.20%
Ca	25.40%
Cl	11.40%
Mg	3.57%
P	3.23%
Si	1.73%
S	1.65%
Fe	0.43%
Al	0.34%
Sr	0.24%
Br	0.14%
Zn	928 PPM

Fig S6. WDXRF (Wavelength Dispersive X-Ray Fluorescence) spectrum showing heavy metal content in leaves of *Prosopis laevigata* plants from Nexapa River region.

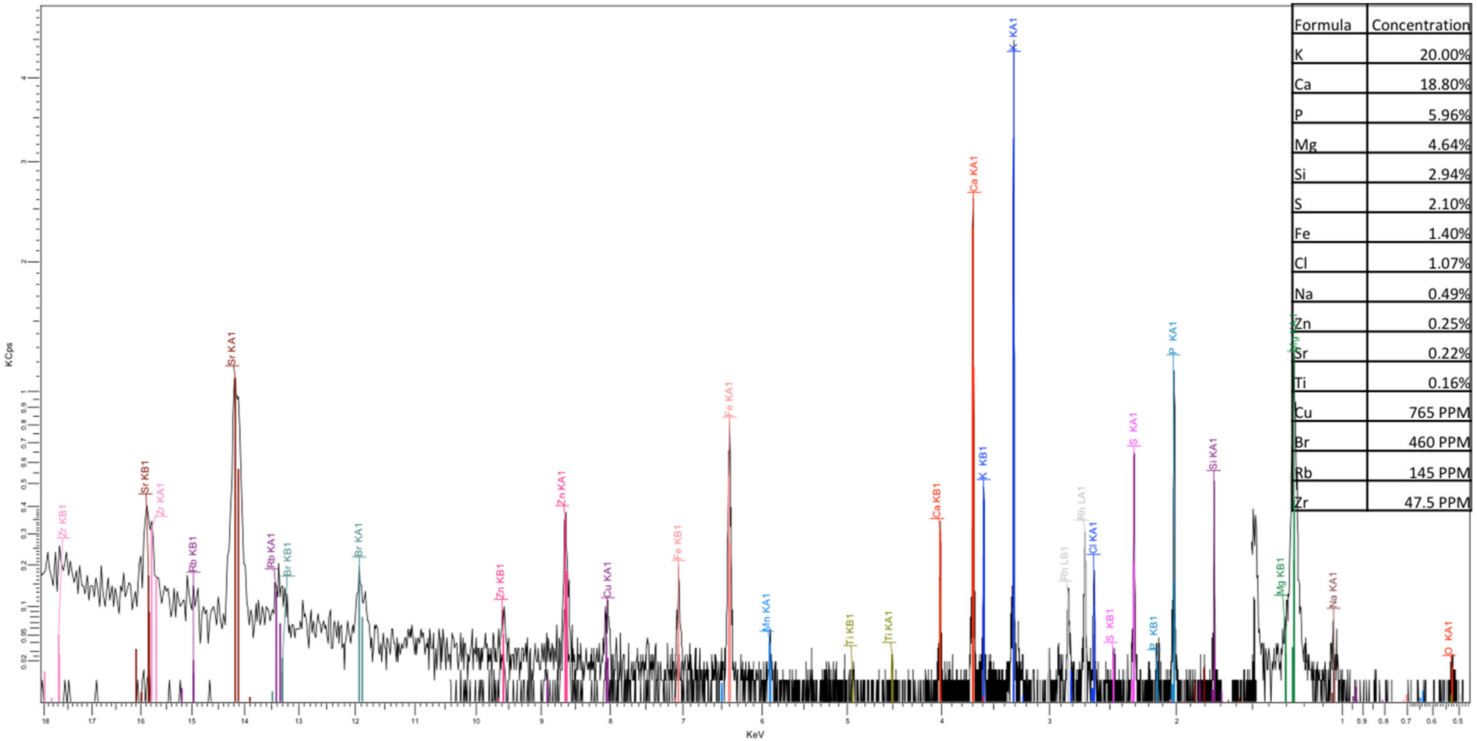

Formula	Concentration
K	20.00%
Ca	18.80%
P	5.96%
Mg	4.64%
Si	2.94%
S	2.10%
Fe	1.40%
Cl	1.07%
Na	0.49%
Zn	0.25%
Sr	0.22%
Ti	0.16%
Cu	765 PPM
Br	460 PPM
Rb	145 PPM
Zr	47.5 PPM

Fig S7. WDXRF (Wavelength Dispersive X-Ray Fluorescence) spectrum showing heavy metal content in pods of *Prosopis laevigata* plants from Nexapa River region.

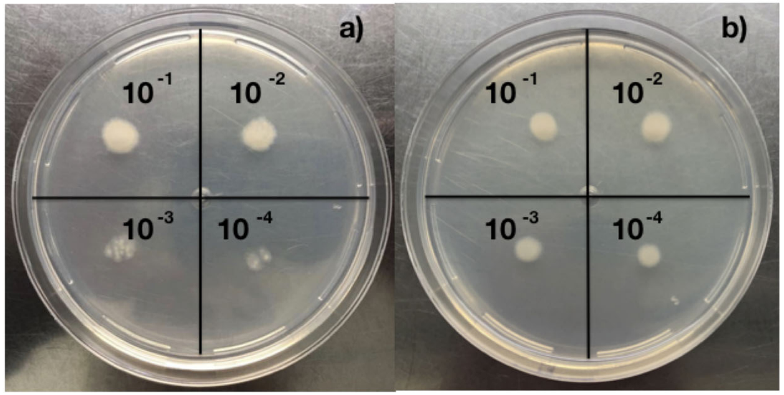

Fig S8. Aluminum tolerance at 10000 mg/kg of *Bacillus* sp. MH778713. a) *Bacillus* sp. MH778713 in YMA medium without aluminum. b) Aluminum tolerance of *Bacillus* sp. MH778713, plated in YMA medium with 10000 mg/kg of aluminum, using serial dilution drop test. MH778713 strain was incubated at 28°C for 48 hours.

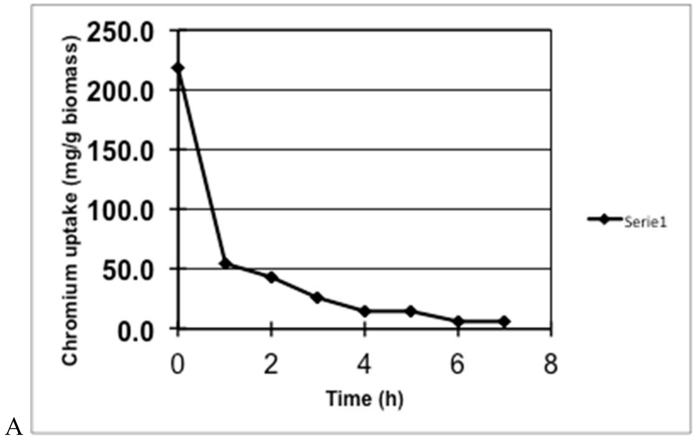

A

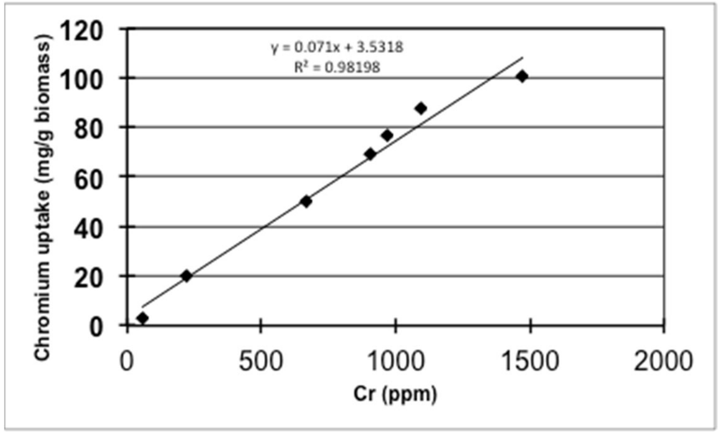

B

Fig S9. A: Environmental chromium uptake at 218 ppm. B: Relationship between initial chromium concentration and maximum metal-uptake by *Bacillus* sp. MH778713**.**

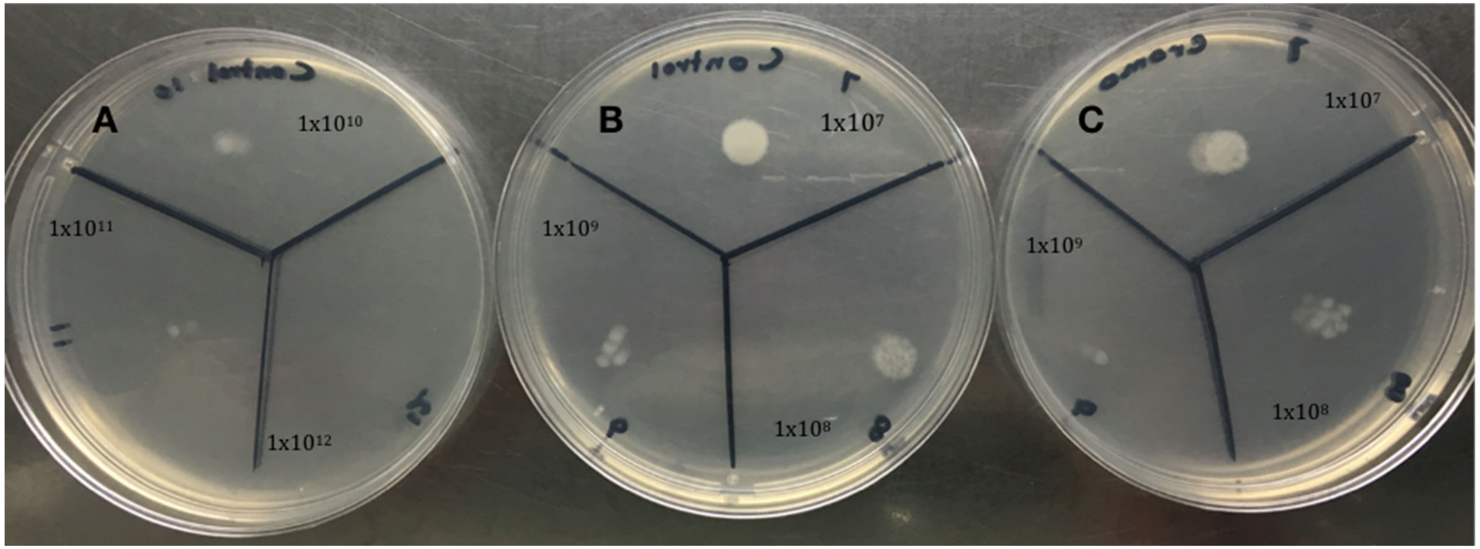

Fig S10. *Bacillus* sp. (MH778713) survival in aqueous solution added with 1474 ppm of chromium (VI). A and B cells at time 0. (C) Cells after 7 hours of exposure at Chromium (VI).
