# Supplementary material for: Chromium Hyper-Tolerant Bacillus sp. MH778713 Assists Phytoremediation of Heavy Metals by Mesquite Trees (Prosopis laevigata)
Source: Front Microbiol. 2019 Aug 13;10:1833. doi: 10.3389/fmicb.2019.01833 (PMC6700308; doi:10.3389/fmicb.2019.01833)
Supplement: Supplementary file 2 [file Data_Sheet_2.pdf]

Table S1. Comparative table showing results of WDXRF (Wavelength Dispersive X-Ray Fluorescence) of sediment, soil and young trees tissues from the Nexapa river region. The concentration of metal is given in mg/kg of dry weight (ppm). Sediment represent samples of Nexapa river sediment, Non rhizospheric soil represents soil samples taken 300 m away from Nexapa river, Rhizospheric soil represent samples of *Prosopis laevigata* rhizospheric soil taken 200 m away from Nexapa river. Reference values are shown from [65].

Element	Sediment (ppm)	Non rhizospheric soil (ppm)	Rhizospheric soil (ppm)	Root (ppm)	Stem (ppm)	Leaf (ppm)	Reference plant tissues values (ppm)	Reference soil values (ppm)
Al	5.29 $x10^4$	4.43 $x10^4$	6.18 $x10^4$	5700	2.06 $x10^4$	4000	$1x10^3$	$1x10^5$
Fe	$4.55x10^4$	4.36 $x10^4$	5.13 $x10^4$	5800	3.2 $x10^3$	4200	1200	500
Ti	5 $x10^3$	4.9 $x10^3$	5.3 $x10^3$	----	2900	-----	80	$9x10^3$
Cr	862	435	208	----	-----	-----	0.2	50
Zn	154	108	174	939	427	<20	20	120
Cu	135	<20	66.9	<20	644	<20	1500	100
Ni	218	<20	-----	----	----	-----	100	90
